# Controlled Synthesis of Dendrite-like Polyglycerols Using Aluminum Complex for Biomedical Applications

**DOI:** 10.1021/acsomega.2c06761

**Published:** 2023-01-04

**Authors:** Govindaraj Perumal, Sreenath Pappuru, Mukesh Doble, Debashis Chakraborty, Shanavas Shajahan, Mohammad Abu Haija

**Affiliations:** † Department of Conservative Dentistry and Endodontics, Saveetha Dental College & Hospital, Saveetha Institute of Medical and Technical Sciences (SIMATS), Chennai600 077, India; ‡ Faculty of Chemical Engineering and the Grand Technion Energy Program, Technion-Israel Institute of Technology, Haifa320003, Israel; § Department of Chemistry, Indian Institute of Technology Madras, Chennai600 036, India; ∥ Department of Chemistry, Khalifa University of Science and Technology, Abu Dhabi127788, United Arab Emirates; ⊥ Center for Catalysis and Separations, Khalifa University of Science and Technology, Abu Dhabi127788, United Arab Emirates

## Abstract

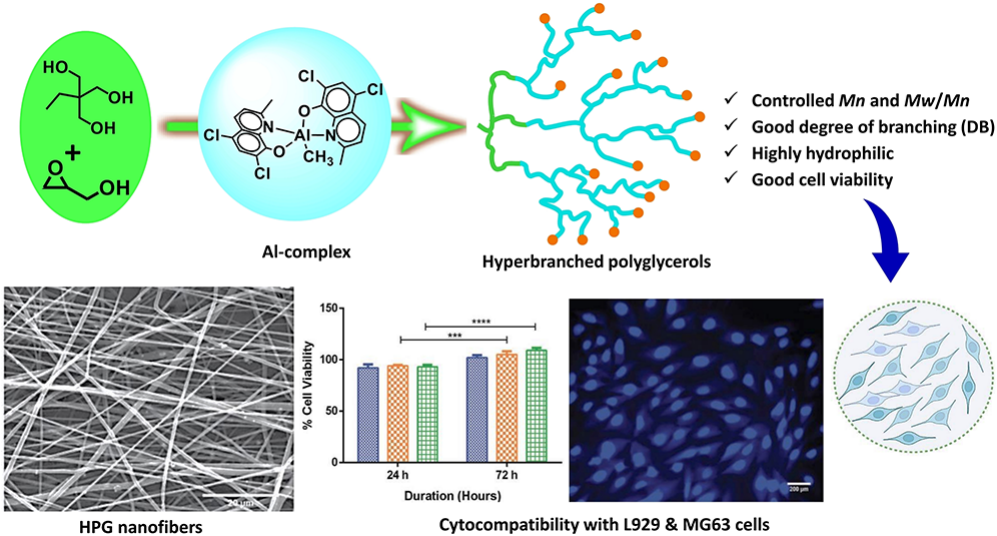

This work describes a one-pot synthesis of dendrite-like hyperbranched polyglycerols (HPGs) via a ring-opening multibranching polymerization (ROMBP) process using a bis(5,7-dichloro-2-methyl-8-quinolinolato)methyl aluminum complex (**1**) as a catalyst and 1,1,1-tris(hydroxymethyl)propane/trimethylol propane (TMP) as an initiator. Single-crystal X-ray diffraction (XRD) analysis was used to elucidate the molecular structure of complex **1**. Inverse-gated (IG)^13^C NMR analysis of HPGs showed degree of branching between 0.50 and 0.57. Gel permeation chromatography (GPC) analysis of the HPG polymers provided low, medium, and high-molecular weight (*M*_n_) polymers ranging from 14 to 73 kDa and molecular weight distributions (*M*_w_/*M*_n_) between 1.16 and 1.35. The obtained HPGs exhibited high wettability with water contact angle between 18 and 21° and *T*_g_ ranging between −39 and −55 °C. Notably, ancillary ligand-supported aluminum complexes as catalysts for HPG polymerization reactions have not been reported to date. The obtained HPG polymers in the presence of the aluminum complex (**1**) can be used for various biomedical applications. Here, nanocomposite electrospun fibers were fabricated with synthesized HPG polymer. The nanofibers were subjected to cell culture experiments to evaluate cytocompatibility behavior with L929 and MG63 cells. The cytocompatibility studies of HPG polymer and nanocomposite scaffold showed high cell viability and spreading. The study results concluded, synthesized HPG polymers and composite nanofibers can be used for various biomedical applications.

## 1 Introduction

Hyperbranched polyglycerols (HPGs)/polyols are an important subclass with a dendrite (tree)-like architecture possessing polymers with three-dimensional, highly branched structures, consisting of more hydroxyl groups, which can be used for further functionalization.^1−3^ HPG polymers possess very good physiochemical properties, biocompatibility, and blood compatibility facilitating usage in various biomedical applications.^4−7^ HPGs can be used as an encapsulating agent for a range of molecules/therapeutic agents, drug and gene delivery, regenerative medicine, stem cell delivery,^8^ and antitumor vaccine preparation.^1,9,10^ In vivo studies demonstrated that HPGs also have similar safety profile characteristics to those of linear poly(ethylene glycols) (PEG);^1,11−13^ hence, they are an appropriate carrier for drug delivery. HPGs consisting of a highly branched architecture with well-separated branches (i.e., no chain entanglements) are very challenging to synthesize.^4^ In 1952, Flory first established a macromolecular polymer with a hyperbranched architecture and polydispersity feature of the HPG polymer.^14^ In 1990, Kim and Webster used the term “hyperbranched polymers” while synthesizing hyperbranched polyphenylene.^15,16^ Hyperbranched polyglycerols were first synthesized by Sandler et al., in 1966, using highly reactive hydroxy epoxides such as glycidol by the ring-opening multibranching polymerization (ROMBP) process.^17^ In the early days of glycidol polymerization, the aim was to obtain linear polymers, but hyperbranched structures were also observed as an undesired side product.^18^ Later, hyperbranched structures were developed using glycidol by cationic polymerization by Penczek and co-workers in 1994.^19^ Recently, Sunder et al. reported controlled synthesis of HPGs by gradually adding glycidol with potassium methylate as a catalyst with a solid-state method and characterized their structure by inverse-gated (IG) ^13^C NMR as an important tool.^20^ Most recently, Ul-haq et al. reported solvent-assisted synthesis of HPGs using glycidol as a monomer and found better polymerization results using 1,4-diaoxane as a good polar solvent. They concluded that the effect of medium also played an important role in controlling the *M*_n_ and MWDs.^21^ Glycidol is a latent AB_2_ monomer, which when added slowly leads to synthesis of hyperbranched polyglycerols through anionic/cationic polymerization.^20^ While synthesizing macromolecular structures with glycidol, a multibranching reaction is achieved by means of deprotonation of its hydroxyl groups.^10^ Many researchers followed this approach with slight modifications in monomers to develop HPGs for biomedical and other applications.^24−29^ However, synthesizing HPGs with controlled molecular weight (*M*_n_), molecular weight distributions (MWDs), and a reasonable degree of branching (DB) is still demanding and needs further optimization in the synthesis procedures and selection of the catalyst. It has been reported that two mechanisms (first with an active chain end and second with an activated monomer) were proposed for the cationic polymerization of glycidol for synthesizing HPGs. Recently, ascorbic acid (Vitamin C) was utilized as an active initiator for the synthesis of low-molecular weight HPGs *via* the activated monomer mechanism.^22^ The most commonly used catalysts for the synthesis of HPGs include alkoxides of potassium, cesium, and di(benzyl)amino ethanol.^10,20,23^ Among these, potassium methylate (KOMe) is extensively used for its better deprotonating ability of the 1,1,1-tris(hydroxymethyl)propane/trimethylol propane (TMP) hydroxyl groups.^21^ However, for biomedical applications, the used catalyst/initiator and solvents for HPG synthesis should be nontoxic and should not induce any adverse effects in the final polymer to be utilized.^24,25^ Interestingly, HPG polymers and/or a combination with other polymers has attracted the attention of many researchers for various biomedical applications such as drug delivery,^26^ multifunctional nanomaterials,^27^ and tissue engineering applications.^28,29^ For example, Kang et al. in 2013 synthesized polycaprolactone (PCL) with an azide end group and coupled it to alkyne-functionalized HPG polymers *via* a Cu(I)-catalyzed alkyne–azide click reaction. The synthesized block copolymers were used to improve antifouling and antibacterial properties.^30^ Also, hyperbranched polyglycerol–poly(lactic acid) (HPG–PLA) electrospun fibers showed improved hydrophilicity and mechanical strength of the composite fibers.^33^ Highly reduced conductive graphene nanoinks (HRG-HPGS) with hyperbranched polyglycerol and PCL nanofibers showed enhanced electrical conductivity and in vitro cell compatibility with stem cells.^27^ HPG polymers were explored for a plethora of biomedical applications, and their possibilities of modification or functionalization with different polymers were described.^28,34−44^

Our earlier studies were carried out with organometallic Nb and Ta complexes as catalysts and TMP as an initiator for the synthesis of HPG polymers by ring-opening polymerization (ROP) of glycidol.^45^ However, because of their weak Lewis acidic nature, we completely failed to achieve high-*M*_n_ HPG polymers at high monomer loadings. In the present work, we described the synthesis and structural characterization of an aluminum complex (**1**) with the 5,7-dichloro-2-methyl-8-quinolinolato proligand (**LH**). The catalytic behavior (1) toward the synthesis of dendrite-like hyperbranched polyglycerols (HPG) using TMP as an initiator at low to high monomer loadings was discussed. The aim of this study is to develop an aluminum complex catalyst to synthesize HPG polymers with different *M*_n_ values and characterize using different analytical techniques including NMR, gel permeation chromatography (GPC), matrix-assisted laser desorption/ionization–time of flight (MALDI-TOF), X-ray diffraction (XRD), Fourier transform infrared spectroscopy (FTIR), water contact angle, and in vitro cytocompatibility studies. In addition to that, we developed nanocomposite fibers using HPG polymers combined with polycaprolactone (PCL) and nanohydroxyapatite (nHA) by an electrospinning process. The fabricated nanofibers were used as a scaffold for tissue engineering applications.

## 2 Materials and Methods

### 2.1 Materials

The chemicals used for Al complex (**1**) preparation, i.e., the ligand 5,7-dichloro-8-hydroxy-2-methylquinoline (**LH**) and trimethylaluminum (AlMe_3_) and deuterated CDCl_3_, were purchased from Sigma Aldrich, India. The trimethylol propane (TMP) initiator, glycidol monomer, polycaprolactone, and nanohydroxyapatite powder were purchased from Sigma Aldrich, India. The monomer glycidol was dried over calcium hydride and distilled twice before use and stored in a glove box. All the chemicals and solvents were stored in the glove box before use unless otherwise mentioned. Dulbecco’s modified Eagle medium (DMEM) and minimum essential medium (MEM) and antibacterial and antifungal mix were purchased from HiMedia, India. Fetal bovine serum (FBS) from Gibco and L929 mouse fibroblast and MG63 human osteosarcoma cells were purchased from the National Center for Cell Science (NCCS), Pune, India. Freshly prepared and dried toluene or tetrahydrofuran (THF) was used by a standard procedure refluxing with sodium/benzophenone. Al complex synthesis was carried out inside the glove box under a dry argon atmosphere. The electrospray ionization (ESI) mass spectrum of the aluminum complex was analyzed with a JEOL GCMATE II GC-MS instrument. The HPG polymerization reactions were also carried out under an argon atmosphere. NMR studies such as ^1^H and inverse-gated ^13^C NMR (IG ^13^C NMR) spectra of the HPG polymers were recorded using DMSO-*d*_6_ as a solvent on a Bruker AVANCE III 500 MHz (AV 500). The molecular weights of HPGs were characterized using a gel permeation chromatography (GPC) system attached with a polySep aqueous GFC column with sodium nitrate (NaNO_3_) solution.^45−47^ The MALDI-TOF spectrum of the polymer was analyzed using a BRUKER, with dihydroxy benzoic acid (DHB) as the matrix. Fourier transform Infrared (JASCO) spectroscopy was used to analyze the functional groups present in the HPGs and nanocomposite fibers (wavenumber region between 400 and 4000 cm^–1^), with a resolution of 4 cm^–1^ with 32 scans. The glass transition temperature (*T*_g_) of the HPGs was analyzed using a DSC instrument (Perkin Elmer DSC 7). The wettability behavior of the sample surface was analyzed using a goniometer (KRUSS, Germany) by measuring the contact angle by introduction of a drop of ultrapure water on six different locations. The nanofiber orientation and morphology and elemental composition were analyzed using a scanning electron microscope with energy dispersive spectroscopy (SEM–EDS) (FEI QUANTA FEG 200, Netherland) after gold sputter coating.

### 2.2 Synthesis and Characterization of the Aluminum Complex (**1**)

**Scheme 1:**
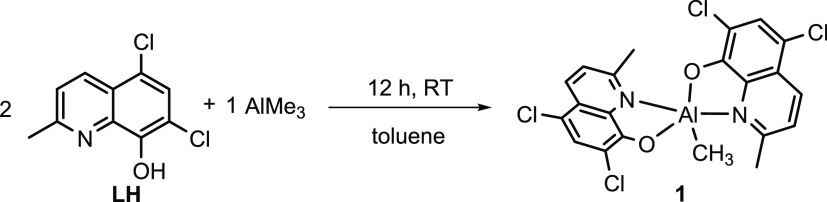
Synthesis of the Aluminum Complex (**1**)

As illustrated in Scheme 1, complex **1** was synthesized by reacting 2 equiv of 5,7-dichloro-8-hydroxy-2-methylquinoline ligand (**LH**) (50 mg, 0.22 mmol) with 1 equiv of trimethylaluminum (AlMe_3_) (0.11 mL, 0.11 mmol) in dry toluene. While adding AlMe_3_, liberation of methane gas was observed, and the resulting reaction mixture was magnetically stirred at 298 K for 12 h, and then, the solvent was removed by vacuum. The obtained solid was washed with hexane, and a yellow crystalline solid of complex **1** was isolated with 80% yield. NMR and ESI data are depicted in Figures S1–S3 in the Supporting Information.

### 2.3 Synthesis of Hyperbranched Polyglycerol (HPG) Using an Aluminum Complex

**Scheme 2:**
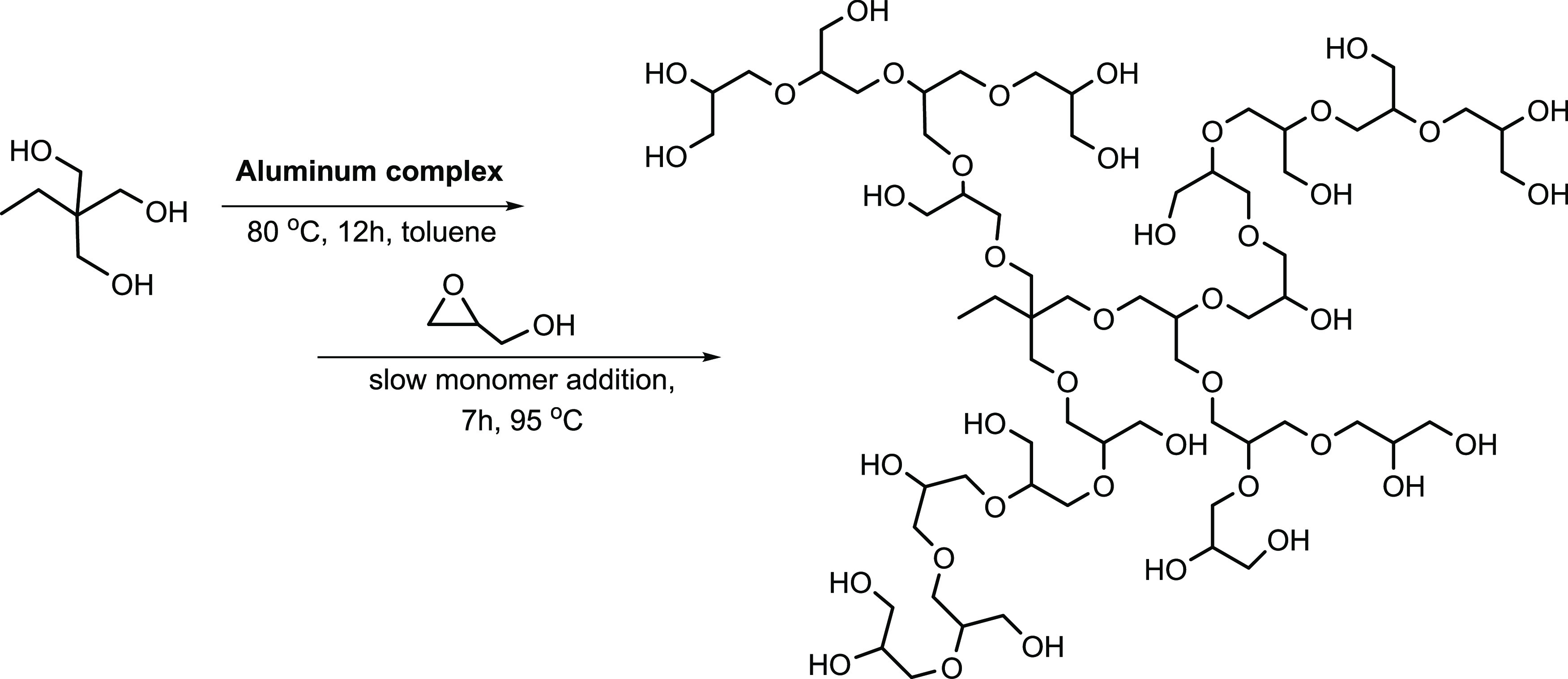
Synthesis Scheme for Hyperbranched Polyglycerols (HPGs)

Multibranched polyglycerols were synthesized by a procedure with slight modifications.^20,47^ As shown in Scheme 2, a predetermined amount of trimethylol propane (TMP) was partially deprotonated using an Al complex at 80 °C for 12 h under dry toluene conditions, Table 1 entry 1, TMP (9.9 mg, 0.074 mmol) and Al complex catalyst (36.7 mg, 0.074 mmol). Next, the calculated amount of glycidol (1 mL, 14.8 mmol for the reaction) was filled carefully into a syringe inside the glove box under an argon atmosphere. Subsequently, the flask containing the above-mentioned mixture of TMP and the Al catalyst was securely sealed with a rubber septum and carefully taken away from the glove box and then fitted with a temperature-controlled (95 °C) oil bath setup along with a magnetic stirrer. The syringe-filled glycidol was very slowly added to a flask containing TMP and the Al catalyst *via* the septum over a period of 7 h by connecting a slow addition syringe pump. After addition, the reaction was continued for another 8 h (total duration of 15 h). After the desired period of time, the reaction was stopped, and the flask containing the resultant solution was fitted in a rotary evaporator to remove toluene. The methanol solvent was used to dissolve the final HPG polymer, and followed by addition of cold acetone, the HPGs were precipitated and filtered. This procedure was carried out 2–3 times; finally, the polymers were subjected to dialysis with deionized water to eliminate low-molecular weight HPGs (in the case of their presence). These purified and vacuum oven-dried (80 °C for 12 h) HPGs were stored carefully for further use. All these polymer samples with different *M*_n_ values were characterized by different physicochemical methods and for in vitro cytocompatibility by cell culture studies.

**Table 1:** Characteristics of Hyperbranched Polyglycerol^a^

entry	M/TMP/Cat.	yield (%)	DB^b^	*M*_n_^c^(kg/mol)	*M*_w_/*M*_n_^c^	no of −OH groups^d^	*T*_g_ (°C)	contact angle (deg)
1	200:1:1	90 ± 2	0.50	14.4	1.16	194	– 45	21
2	400:1:1	87 ± 3	0.50	29.2	1.25	353	– 39	19
3	600:1:1	91 ± 1	0.56	43.2	1.19	366	– 42	18
4	800:1:1	90 ± 2	0.58	57.6	1.30	377	– 55	18
5	1000:1:1	88 ± 2	0.57	73.5	1.35	407	– 40	18

a M/TMP/Cat = glycidol:TMP:Cat. molar ratio.

b Degree of branching (DB) obtained from IG ^13^C NMR studies.

c Obtained from GPC using the NaNO_3_ eluent and poly(ethylene glycol) standard.

d Calculated using proton NMR studies.

### 2.4 General Procedure for the Preparation of the Polymer Blends and Nanocomposites

PCL beads (*M*_w_: 73,000–80,000) were dissolved in dimethylformamide (DMF) and CHCl_3_ (solvent ratio of 1:3 to obtain 10 wt % of the polymer solution). Separately, 20 w/v % of HPG polymer (14 kDa) and nanohydroxyapatite (nHA) powder (10 wt %), respectively, were mixed in DMF under a water bath sonicator for 10 min and then added very slowly to the PCL/HPG polymer blend solution and again sonicated for 10 min and kept overnight with magnetic stirring to obtain an electrospinnable homogeneous composite solution. This nanocomposite was denoted as PHNH unless otherwise mentioned [P: PCL (polycaprolactone), H: HPG (hyperbranched polyglycerol), and NH: nHA (nanohydroxyapatite)].

### 2.5 Development of Nanocomposite Electrospun Fibers

The electrospinning procedure was carried out as per our earlier reported procedure with slight modifications.^28,48^ The nanofibers collection time was carried out for 2 h, subsequently the deposited electrospun fibers of PCL and PCL/HPG/nHA (PHNH) from the collector plate were carefully detached. The fabricated electrospun nanofibers was kept overnight in a vacuum desiccator for drying. The developed scaffolds were analyzed using SEM–EDS and cell culture studies.

### 2.6 In Vitro Cell Viability (MTT) Assay

The percentage (%) cell viabilities of HPG polymers and electrospun nanocomposite samples were determined with L929 and MG63 osteosarcoma cells, respectively. L929 and MG63 cells were inoculated using media containing Dulbecco’s modified Eagle medium (DMEM) and MEM, respectively, 10% fetal bovine serum (FBS), and antibiotic–antimicotic mix and incubated at 37 °C with 5% CO_2_. HPG polymers and electrospun nanofibers with a concentration of 0.2 g/mL and 1x1 cm^2^ respectively. DMEM and MEM media, respectively, were added into the tissue culture polystyrene plate and incubated for 24 h to get the extracts of each sample (PCL and PHNH) used for cell culture studies. L929 and MG63 cells with a density of 1 × 10^4^ cells/well were inoculated and incubated for 24 and 72 h respectively.^28,49,50^ After the specified duration, MTT solution 20 μL (0.5 mg/mL) was replaced to all the wells and further incubated for 3–4 h. The optical density (OD) was recorded at 570 nm with the help of a multimode spectrophotometer (Enspire). Finally, the percentage (%) of viable cells was quantified using the formula OD of test/OD of control.

### 2.7 Cell Morphology Staining with Electrospun Nanocomposite Scaffolds

MG63 cells were seeded to different wells (1 × 10^4^ cells/well) of a 24-well TCPS plate and incubated for 1 d for attachment of the cells. Then, all the wells containing media were changed with 500 μL of the sample-extracted media (collected as mentioned in the previous section) except the control wells. Subsequently, they were incubated for 12 h, and then, medium was replaced and washed with phosphate-buffered saline (PBS) to remove the nonadhered cells. Each well containing cells were fixed (15 min) by adding 4% of paraformaldehyde and washed 2–3 times with PBS. Cells were permeabilized for 5 min by addition of Triton X-100 (0.5%) dissolved in PBS and washed again with PBS 2–3 times. The nuclei and cytoskeleton were stained with DAPI (Sigma Aldrich) and FITC (Fluorescein isothiocyanate), (Medox India) respectively. The cell nuclei and cytoskeleton were observed under a fluorescence microscope (Olympus IX71, Japan).

## 3 Results and Discussion

The present study involves the first-time synthesis of HPGs using a bis(5,7-dichloro-2-methyl-8-quinolinolato)methyl aluminum complex as a catalyst^51^ and TMP as an initiator. Previously, potassium alkoxide,^5,20,46,47,52−55^ potassium tertiary butoxide,^23,56,57^ cesium alkoxide,^58,59^ and di(benzyl)amino ethanol^60^ were used as a catalyst/initiator for synthesizing HPGs. Among them, KOMe is extensively used due to its deprotonating ability of the hydroxyl groups of TMP.^21^ Herein, we proposed a new approach of using an aluminum complex catalyst (**1**) for deprotonating the hydroxyl groups of TMP with enhanced control over HPG synthesis and at the same time without compromising the inherent properties of the polymer for biomedical applications. The aluminum complex exhibited better catalytic activity with respect to that of the control in *M*_n_ and MWDs (1.16–1.37) and comparable to that of earlier mentioned initiators/catalyst for ROP glycidol and synthesizing hyperbranched polyglycerols (HPGs). Herein, we elaborated on the preparation method of the aluminum complex (**1**) and its single-crystal X-ray characteristics. It should be noted that this is the first report explaining an aluminum metal complex as a catalyst for synthesizing HPGs with low to high *M*_n_ and controlled MWDs. The polymerization results of the synthesized HPGs with different concentrations of glycidol and TMP initiators are listed in Table 1.

**Figure 1:**
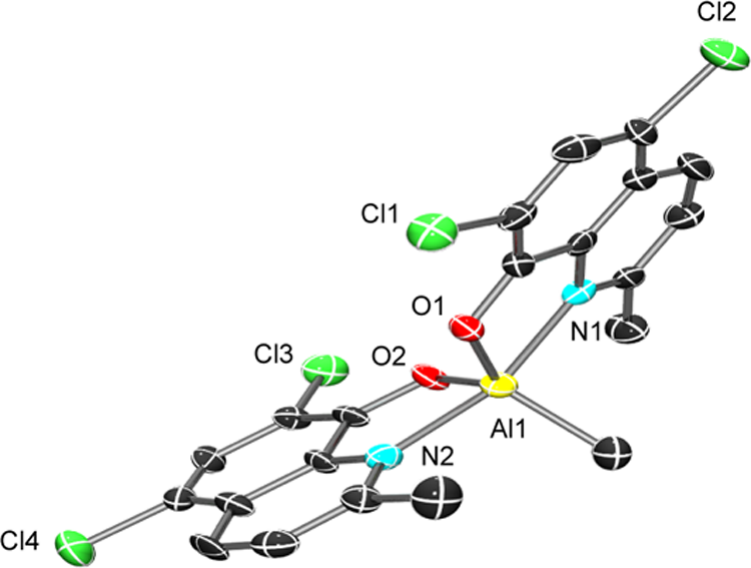
ORTEP (30% probability) of **1**. Chosen bond distances and bond angles (Å, deg) of the synthesized complex **1**: Al1-O1 = 1.7789(2), Al1-O2 = 1.7568(3), Al1-N1= 2.0944 (3), Al1-N2 = 2.0731 (3), O1-Al1-N1 = 100.72(12), and O2-Al1-N2 = 155.53(10).

The ligand 5,7-dichloro-8-hydroxy-2-methylquinoline (**LH**) used in this work was a commercially available compound. Recently, Williams and co-workers reported a bis(5,7-dichloro-2-methyl-8-quinolinolato)ethyl aluminum complex as a catalyst for *rac*-lactide (*rac*-LA) polymerization for the synthesis of isotactically rich poly(lactide) (*P*_i_ = 0.76).^51^ Furthermore, in the previous reports, aluminum quinolate complexes were reported as attractive catalysts for ROP of cyclic esters.^31,32^ In the present work, the formed single crystals of complex **1** (aluminum complex) were carefully picked from dry toluene and subjected for single-crystal XRD analysis. The ORTEPs corresponding to the Al complex (CCDC number: 1443888) are depicted in Figure 1, along with the selected bond lengths and angles. The complex has a pentacoordinate metal center with a distorted trigonal bipyramidal coordination environment. As shown in Figure 1, because of the less steric influence from the ligand, the bis(ligated)aluminum methyl complex structure was observed. Metal and phenolate oxygen bond lengths are 1.7789(2) and 1.7568(3) Å, respectively, and metal–nitrogen bond lengths are 2.0944(3) and 2.0731(3) Å. The O–M–N bond angles are 100.29(9)° [O1-Al1-N1]^1,10^ and 100.29(9)° [O2-Al1-N2]. The bond lengths and angles were well supported by the literature data.^51^ The crystallographic data are depicted in the Supporting Information (Table S1).

### 3.1 Structural Characterization of HPG Polymers

**Figure 2:**
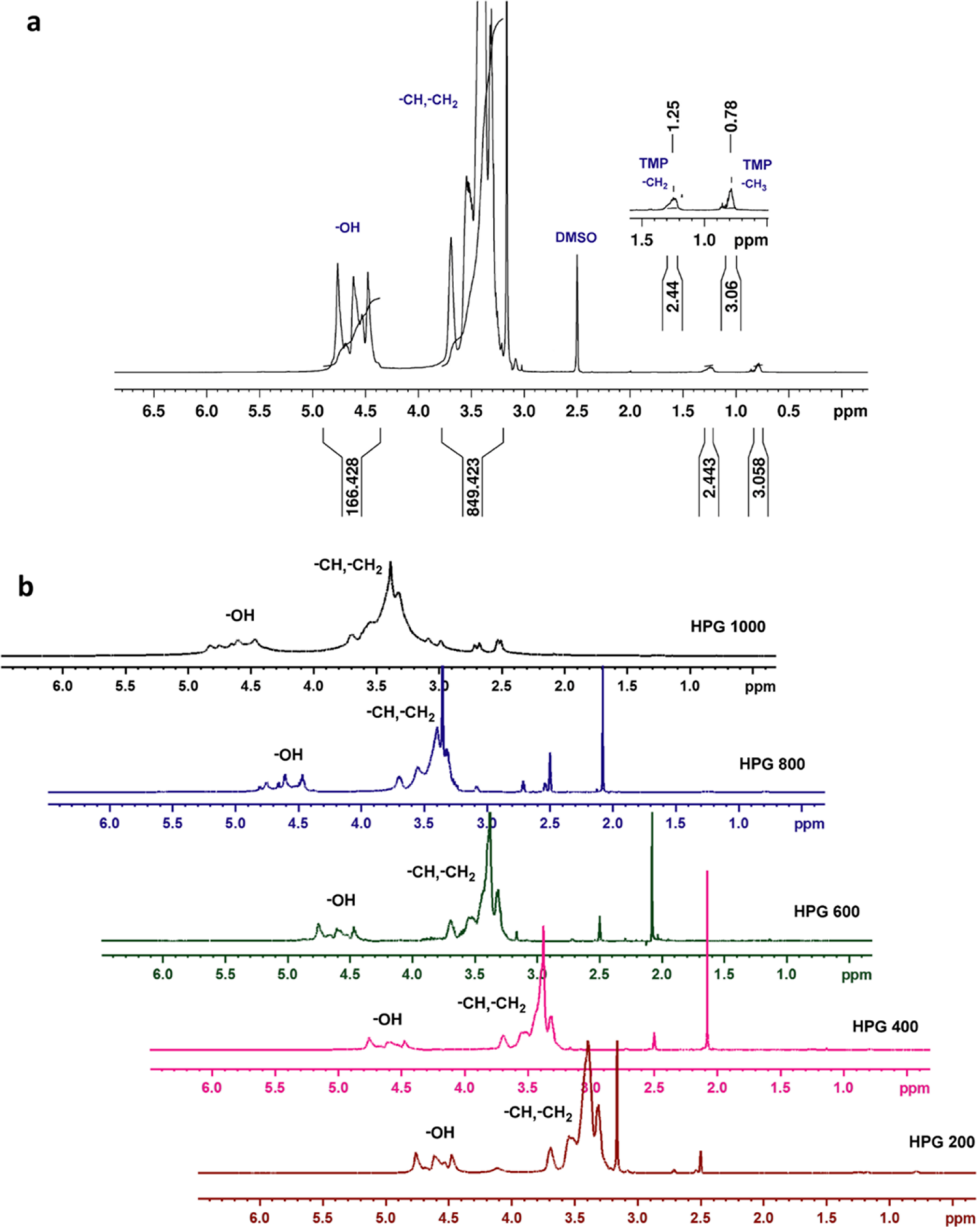
(a) ^1^H NMR spectrum of hyperbranched polyglycerol and (b) ^1^H NMR spectra of hyperbranched polyglycerols synthesized with different ratios of monomers.

^1^H NMR is used to validate the incorporation of the TMP initiator into glycidol (Figure 2a). The presence of methyl and methylene groups of TMP at 0.8 ppm (−CH_3_) and 1.2 ppm (−CH_2_) in ^1^H NMR confirms the incorporation of TMP. As shown in Figure 2a, methylene and methine resonances for the polyether backbone of HPGs were observed between 3.2 and 3.8 ppm with the presence of a broad signal between 4.4 and 4.8 ppm assigned to the terminal hydroxyl groups of the HPGs. The comparative ^1^H NMR spectra of HPGs with different monomer-to-catalyst ratios are shown in Figure 2b. All these spectra reveal the same peak shift values, and the number of terminal hydroxyl groups present in each HPG is calculated using the proton NMR, and the values are listed in Table 1. Increasing the monomer-to-catalyst ratio increases the number of terminal hydroxyl groups, and one can easily add the desired functional groups for precise applications.

**Figure 3:**
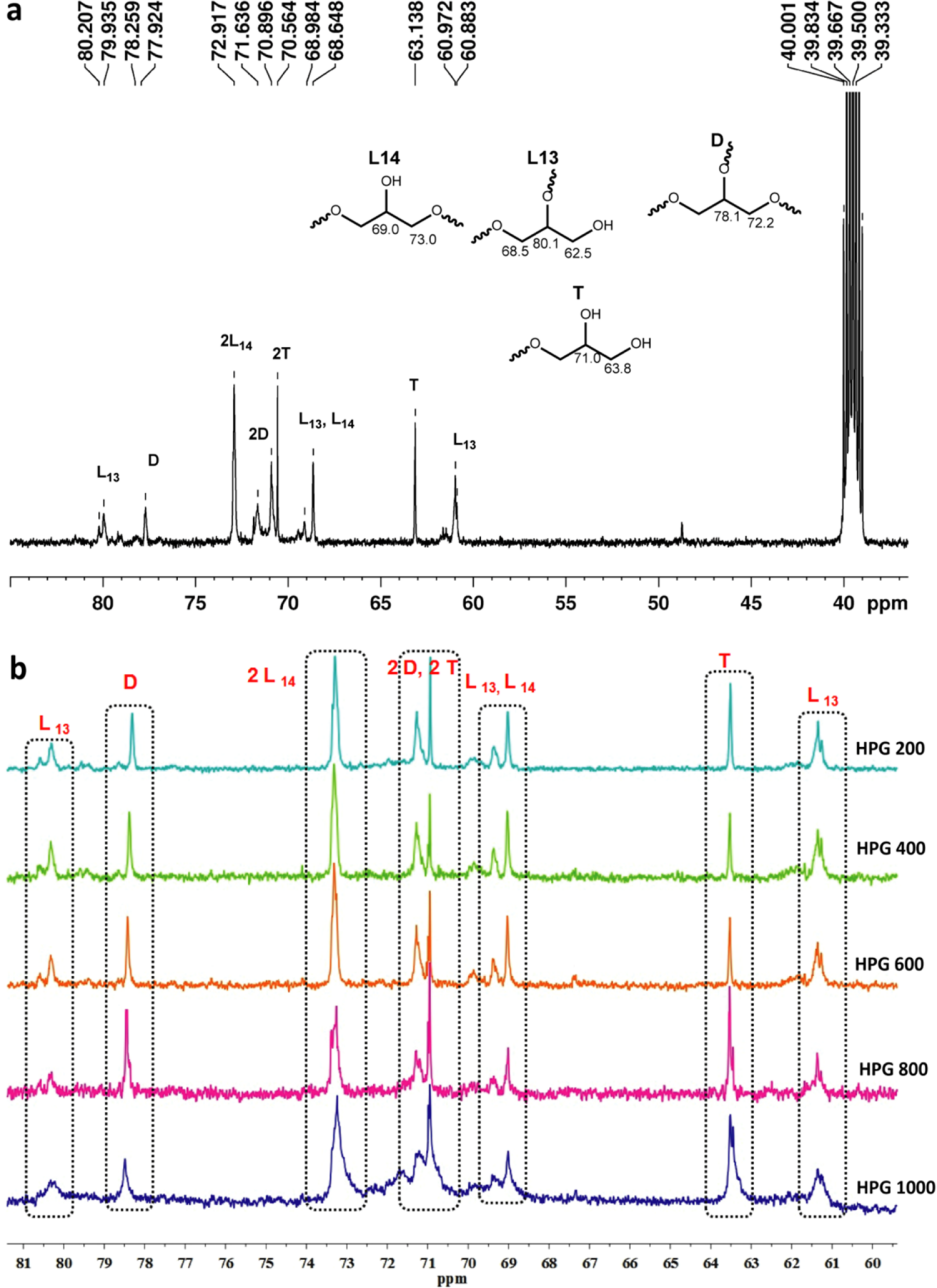
(a) Inverse-gated (IG)^13^C NMR spectrum of hyperbranched polyglycerol and (b) comparison of IG ^13^C NMR spectra of hyperbranched polyglycerols at different ratios of monomers.

Inverse-gated (IG) ^13^C NMR studies are utilized to obtain in-depth information about HPGs, and it is used to calculate the degree of branching (DB).^20^ The spectrum indicated the presence of seven distinctive peak shift values between 60 and 85 ppm corresponding to the hyperbranched structures (Figure 3a), and comparative (IG) ^13^C NMR spectra of HPGs with different monomer-to-catalyst ratios are shown in Figure 3b. Similar peaks were observed in the previously reported literature.^20,28,45^ The structure of the hyperbranched polymers lies between the conventional linear and dendritic forms.^4^ However, polymers having a DB of 0 are noted as linear structures with those of 0.50–0.66 termed as hyperbranched and those having a DB of 1 called as dendrimers.^60^ By calculating the relative abundance percentage (%) from the signal intensities of the IG ^13^C NMR spectrum, the DB can be derived from the following equation^20^
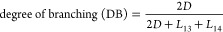
where *L*_13_ = linear 1, 3; *L*_14_ = linear 1,4; *D* = dendritic, and *T* = terminal units.

These structural units are characterized as *L*_13_, *L*_14_, *D*, *T*, 2*D*, and 2*T* units (Figure 3a). The structural components of HPGs having one methine and two methylene carbons were determined, also to evaluate the units present in terminal positions which are equal to the number of hydroxyl groups and dendritic units.^20^ The DB of the synthesized HPG polymers showed a value of 0.50–0.58 with well-controlled MWDS (≤1.35), as shown in Figure 3a,b. This indicates that the present single-site aluminum catalyst has good control over the *M*_n_ and MWDs.

### 3.2 Gel Permeation Chromatography (GPC) and MALDI-TOF Analysis of HPG Polymers

**Figure 4:**
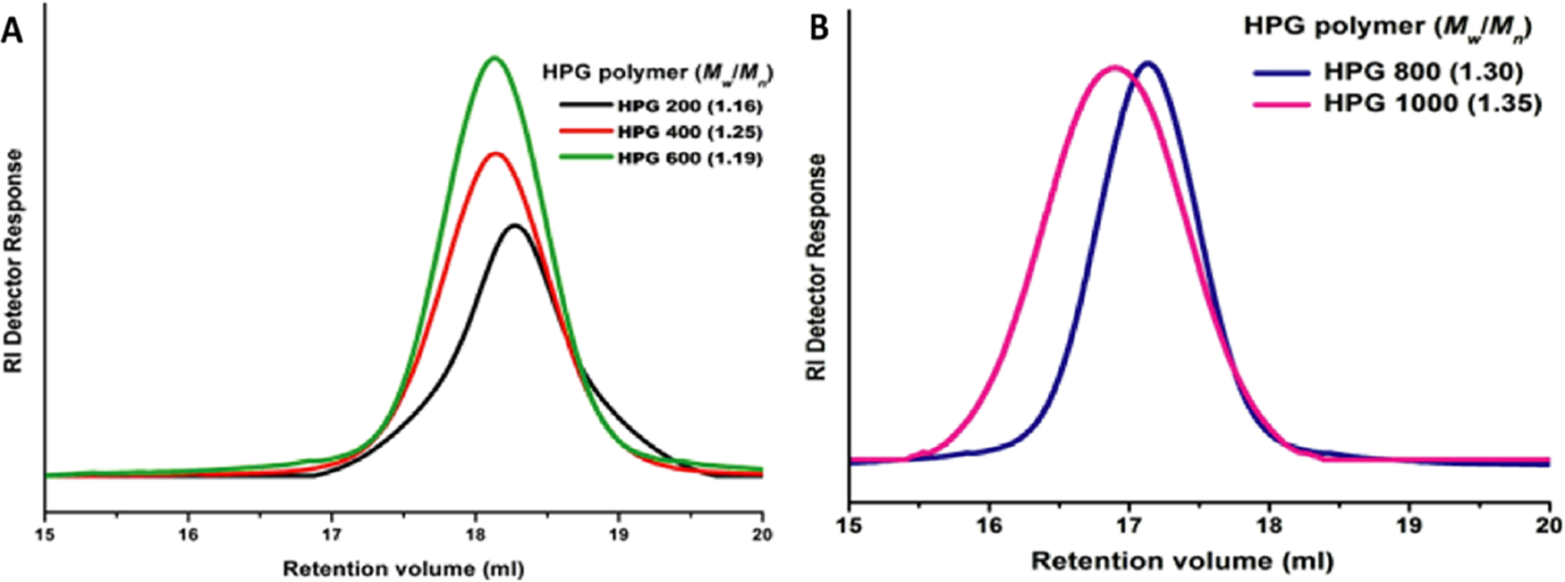
(A) and (B) GPC chromatograms of HPG polymers (200–1000 ratio) of low and high *M*_n_ using the catalyst and glycidol at various ratios.

GPC revealed that the molecular weights (*M*_n_) of the polymer increase with increasing monomer-to-catalyst ratios, while the molecular weight distribution remains constant with unimodal distribution. The GPC chromatograms showed monomodal distributions (Figure 4A,B). The absence of entanglement between the chains was confirmed from the observed narrow polydispersity index (PDI) values; if the PDI is broad, more side chains and chain entanglements could be possible.^4^ The end group analysis of the MALDI-TOF spectrum of low-molecular weight HPGs (Figure S4, Supporting Information) indicates that TMP was incorporated into the HPG polymer. In addition, the incorporation of the TMP initiator into the polymer chain was further confirmed from oligomer ^1^H NMR (Figure S5, Supporting Information). All MALDI ionization peaks showed the exact molecular weight differences of glycidol.^58^ In addition to this narrow distribution determined by GPC, a good agreement regarding the sequences of the polymer chain was also observed from MALDI-TOF analysis.

### 3.3 Differential Scanning Colorimetry (DSC) and FTIR Analysis of Hyperbranched Polyglycerols

From the DSC analysis, the HPG glass transition temperatures (*T*_g_) are recorded between −38 and −55 °C (Figures S6–S10, Supporting Information). The polymers physically change from oily to the solid phase as the molecular weight of the polymer increases.^20^ FTIR spectroscopy is used to determine the functional groups present in the polymers. A characteristic broad absorption peak at 3470 cm^–1^ corresponding to the stretching of hydroxyl (−OH) groups is observed (Figures S11–S15, Supporting Information). This absorption peak area increases with increasing monomer-to-catalyst ratio. Characteristic absorption peaks at 2992, 1990, 1653, and 1056 cm^–1^ for −CH_2_, −CH, −C–C, and C–O–C stretching, respectively, are also observed; this clearly demonstrates the multibranching nature and the presence of more terminal hydroxyl groups in the HPGs.

### 3.4 Water Contact Angle Measurement of HPG Polymers

**Figure 5:**
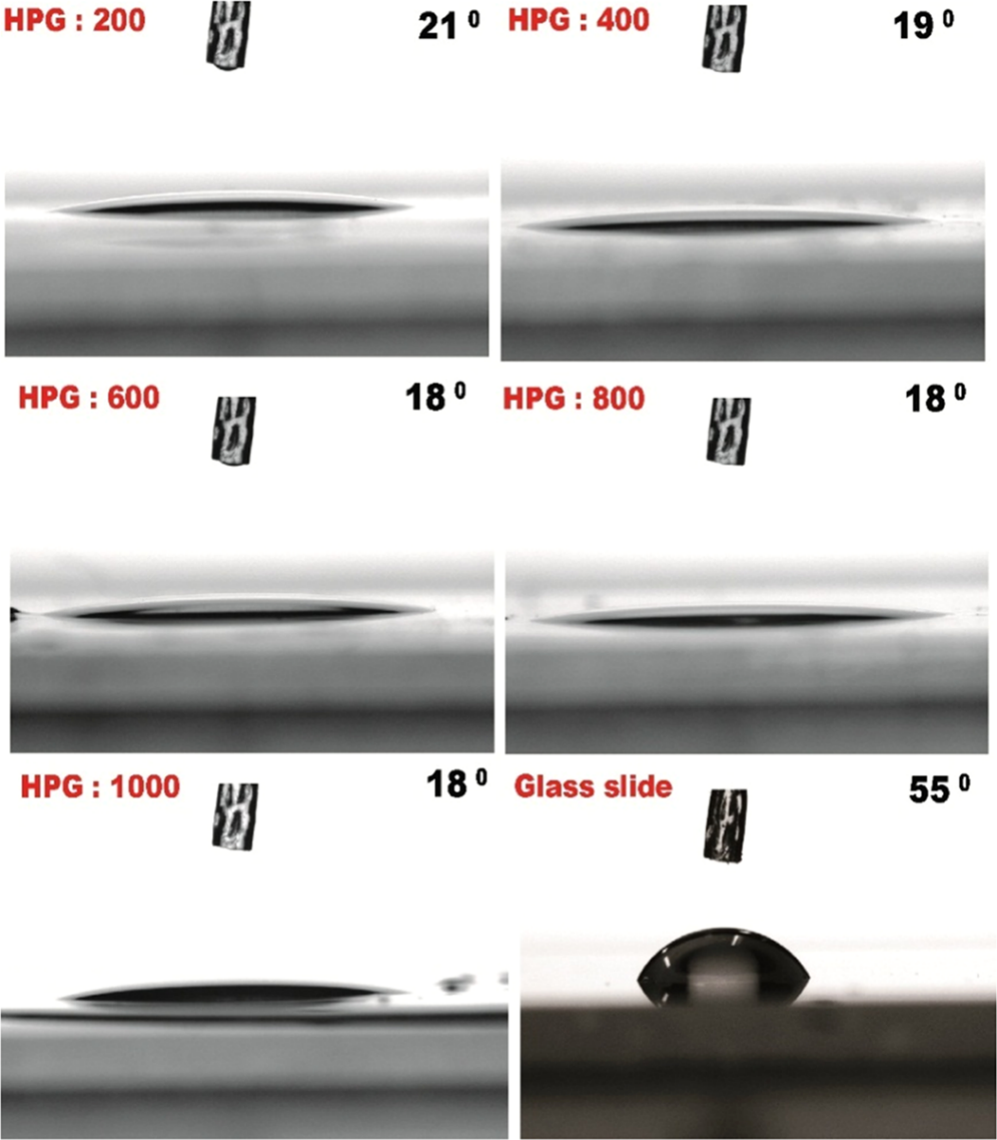
Water contact angle measurement of HPG polymers coated on a glass slide and uncoated glass slide (control).

The high hydrophilic nature of these synthesized polymers was determined using water contact angle analysis. Wettability of HPGs increases with increasing molecular weight (Figure 5), due to more intermolecular interactions of the terminal hydroxyl groups as reported earlier.^53^ The water contact angle of HPGs was shown to be between 18 and 21°. By increasing the glycidol ratio, the number of hydroxyl groups increases due to the broadening in the proton NMR resonance values from 4.4 to 4.8 (Figure 2B). Hydrophilicity is a key property of any material used for biomedical applications such as drug delivery carriers, multifunctional attachment sites for therapeutic agents, contrast agents for imaging, protein delivery, biomineralization, tissue engineering, etc.^4^ HPG polymers are highly suitable for drug delivery applications due to their higher solubility in the aqueous phase, no or less chain entanglements, well-compact and three-dimensional (3D) macromolecular structure, high flexibility, less viscosity, and enormous free hydroxyl groups at terminals, favoring multiple derivatives or attachment possibilities.^54^ The improved hydrophilic nature of HPGs exhibits many significant characteristics such as nonspecific protein adsorption prevention, antifouling nature, and superior thermal behaviors compared to those of linear poly(ethylene glycol) (PEG).^53^ HPG polymers are biocompatible due to their biochemical nature and the presence of glycerol, which is involved in the citric acid cycle.^54^ Several reports suggested that HPGs demonstrate very good biocompatibility and thus are exceptionally valuable in biomedical applications.^46^ In vitro cell viability assay was performed to determine the noncytotoxic nature of HPG polymers against L929 cells over a period of 72 h, which indicates that there is no sign of cytotoxicity and higher cell viability is present.

### 3.5 Scanning Electron Microscopy (SEM) Analysis of the HPG Blend Nanocomposite

**Figure 6:**
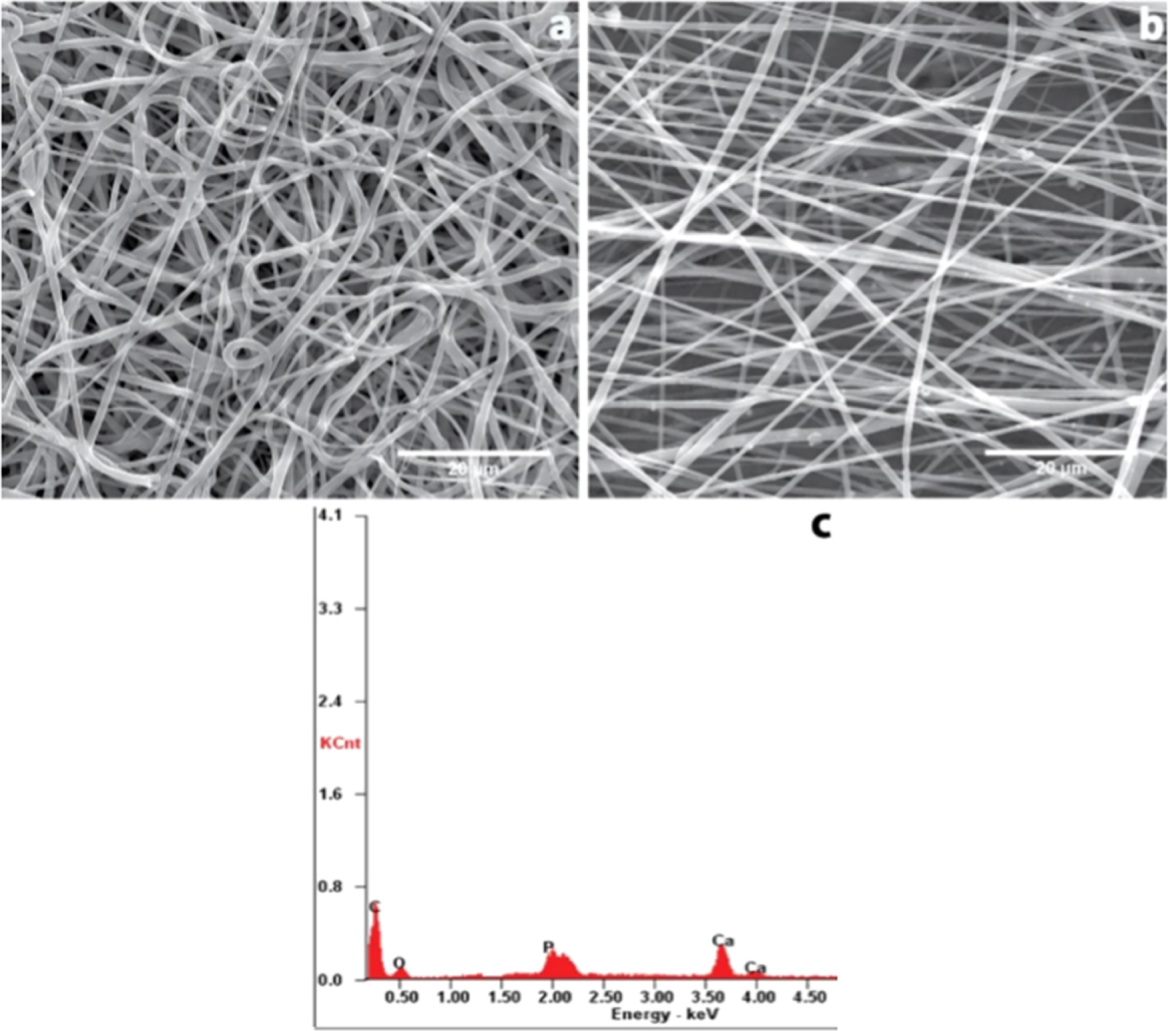
SEM images of electrospun nanofibers containing (a) PCL and (b) PHNH nanofiber scaffolds with uniformly dispersed nanohydroxyapatite (nHA) and (c) energy dispersive spectrum (EDS) of the PHNH nanocomposite showing the elemental composition (Ca^2+^, P, O, and C).

Electrospun nanofibers observed under SEM showed PCL and PHNH (PCL-HPG polymer blend containing nanohydroxyapatite) bead-free randomly aligned nanofibers (Figure 6). The PHNH nanocomposite showed the incorporation of calcium phosphate within the fibers. The average fiber diameter of PCL and PHNH scaffolds was observed to be 653 and 521 nm, respectively. In addition to that, PHNH scaffold nanofibers were well-oriented in a direction than those of PCL. From our earlier reported studies, the addition of HPG to the polymer blend improves the smoothness of the fibers and orientation with reduced fiber diameter and bead-free fibers nonetheless at higher concentrations.^28^

### 3.6 In Vitro Cell Viability (MTT) Assay

**Figure 7:**
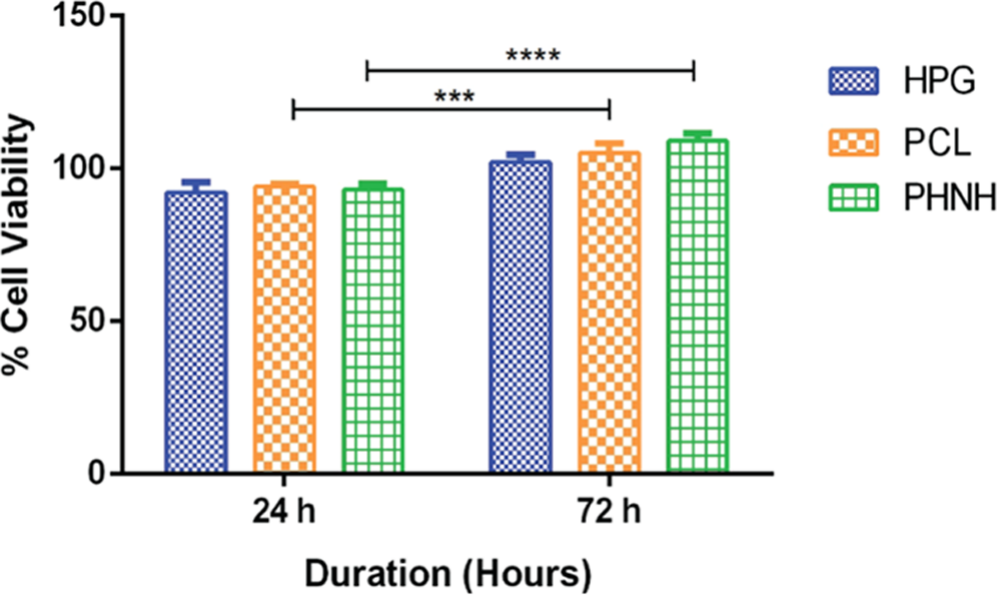
In vitro cell viability L929 cells with the HPG polymer and MG63 cells with PCL and PHNH electrospun scaffolds for the durations of 24 and 72 h.

HPG polymers have very good biocompatibility due to their biochemical nature and are present in the body as glycerol, which is involved in the citric acid cycle.^54^ Several reports suggested that HPGs demonstrate very good biocompatibility and so are exceptionally valuable in biomedical applications.^29,46,54^ MTT assay was performed to show the in vitro cell viability of HPG polymers using L929 cells for the durations of 24 and 72 h (Figure 7). MTT assay is an indicator of the number of viable cells and shows the mitochondrial function of the live cells. This study revealed that HPG polymers show very good cytocompatibility over 72 h of incubation. No sign of cell toxicity was observed with HPG polymers using L929 cell lines. This undoubtedly demonstrates that HPG polymers synthesized using the aluminum complex initiator do not exert significant toxicity and hold any residual catalyst, solvent, and monomers. Similarly, cell viability with MG63 cells with PCL and PHNH electrospun scaffolds was also determined for the durations of 24 and 72 h. Higher cell viability with PHNH scaffolds was observed when compared to that of PCL electrospun nanofibers. This improvement in cell viability was attributed to the addition of the HPG polymer and calcium phosphate in PHNH scaffolds unlike PCL scaffolds. This improved cell viability of the PHNH scaffold makes it an ideal biomaterial for tissue regeneration applications.

### 3.7 In Vitro Cell Adhesion Studies

**Figure 8:**
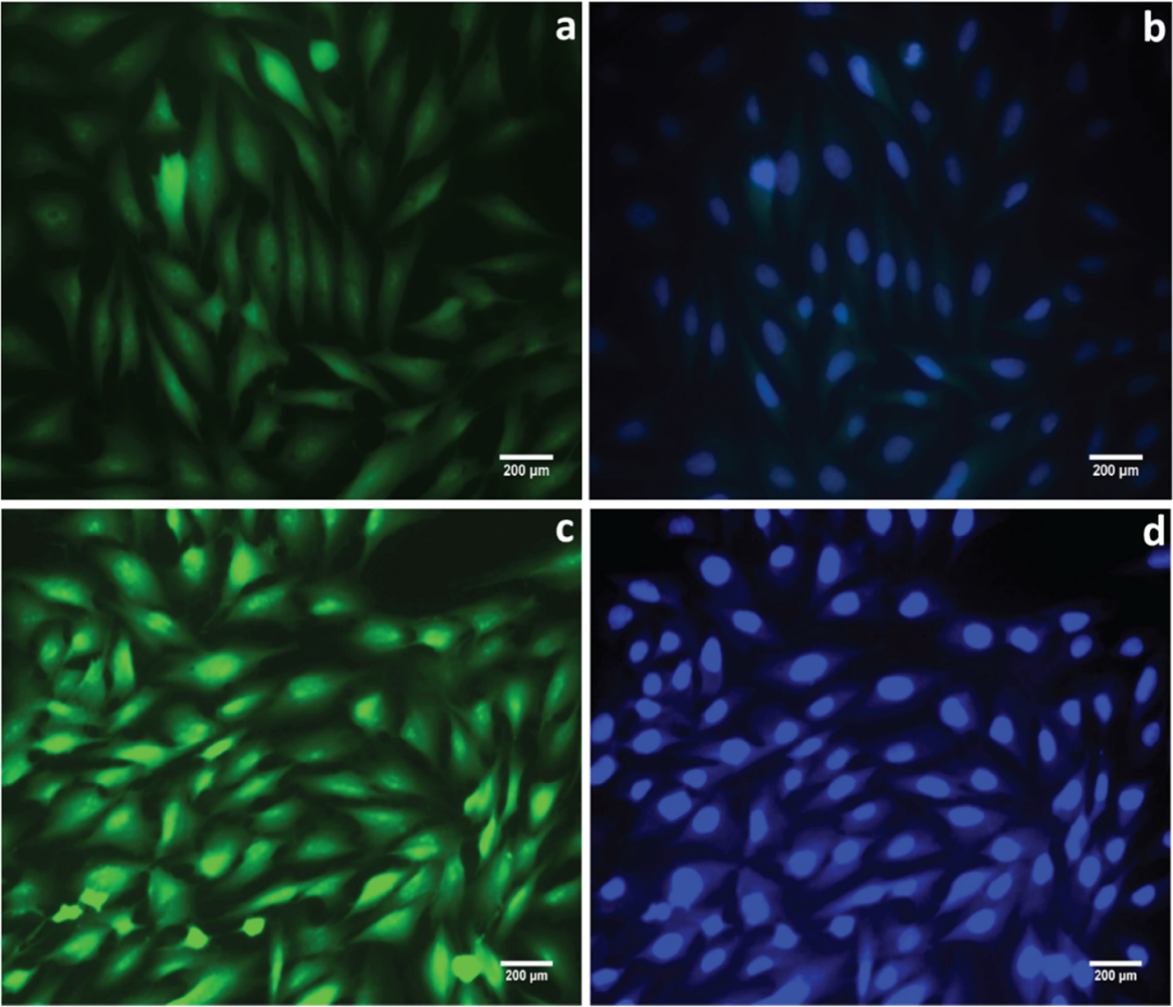
Fluorescence microscopy images of MG63 cell adhesion and proliferation with PCL electrospun nanofibers (a, b) and (c, d) HPG blend nanocomposite (PHNH) [FITC is used for staining the cytoskeleton (green), and DAPI is used for staining the nucleus (blue)].

Cell adhesion studies were carried out with electrospun nanofibers containing PCL and PHNH extracts incubated for 24 h (Figure 8). Superior cell adhesion, filopodium extension, and actin cytoskeleton spread of the MG63 cells were observed with PHNH scaffold extracts compared to those of PCL. This improved cell adhesion and spreading were also evidenced by cell viability assay results with PHNH scaffolds. This indicates that the nanocomposite scaffold PHNH can favor the initial adhesion and proliferation of the osteoblast-like cells, which improve the scaffold bioactivity.

## 4 Conclusions

The present study demonstrated the successful synthesis and characterization of hyperbranched polyglycerols using a single-site aluminum complex (**1**) as a catalyst and TMP as an initiator. Single-crystal XRD study has confirmed the molecular structure of the bis(ligated)aluminum methyl complex (**1**). GPC analysis of HPGs showed well-controlled molecular weight (*M*_n_) and molecular weight distribution, *M*_w_/*M*_n_ (≤1.35). Particularly, ^13^C IG NMR revealed a very good degree of branching (0.50–0.57) due to the use of the single-site active aluminum complex and slow addition of glycidol. The synthesized HPG polymers were also blended with PCL and nanohydroxyapatite to develop electrospun nanocomposite scaffolds for tissue engineering applications. The synthesized HPG polymers were extremely hydrophilic and did not exhibit cytotoxicity toward L929 cells. The results described here are the first examples of ROP of glycidol using Al complex as a catalyst. The PHNH nanocomposite scaffolds showed well-dispersed nHA particles within the nanofibers. The developed nanocomposite scaffold exhibited high cell viability, cell adhesion, and spreading with MG63 cells. Hence, HPG polymers and their nanocomposites scaffolds are potential scaffolds for various biomedical applications.

## Supporting Information Available

^1^H NMR (400 MHz, CDCl_3_) of the Al compound, **1**; ^13^C NMR (100 MHz, CDCl_3_) of the Al compound, **1**; ESI-mass spectrum of the Al compound, **1**; MALDI-TOF spectrum of low-molecular weight HPGs using the aluminum complex as a catalyst and TMP as an initiator; ^1^H NMR spectra (400 MHz, CDCl3) of only the 1,1,1-tris(hydroxymethyl)propane (TMP) initiator and TMP-reacted aluminum complex in 1:1 molar ratio and oligomer species prepared using the TMP-reacted aluminum complex (1 equiv) and glycidol (5 equiv) in dry toluene at 95 °C, 2 h; DSC thermogram of HPG 200–1000; FTIR spectrum of HPG 200–1000; and crystallographic data for complex **1** (PDF)
