# Supplementary material for: Controlled Synthesis of Dendrite-like Polyglycerols Using Aluminum Complex for Biomedical Applications
Source: ACS Omega. 2023 Jan 4;8(2):2377–88. doi: 10.1021/acsomega.2c06761 (PMC9851026; doi:10.1021/acsomega.2c06761)
Supplement: Supplementary file 1 — ao2c06761_si_001.pdf [file ao2c06761_si_001.pdf]

Supporting Information

Controlled synthesis of dendritic like polyglycerols using aluminium complex for biomedical applications

Govindaraj Perumal[1, †,*], Sreenath Pappuru[2,†,*], Mukesh Doble[1], Debashis Chakraborty [3], Shanavas Shajahan [4], and Mohammad Abu Haija[5]

[1]Department of Conservative Dentistry and Endodontics, Saveetha Dental College & Hospital, Saveetha Institute of Medical and Technical Sciences (SIMATS), Chennai - 600 077, India.

[2]Faculty of Chemical Engineering and the Grand Technion Energy Program, Technion-Israel Institute of Technology, Haifa - 320003, Israel.

[3]Department of Chemistry, Indian Institute of Technology Madras, Chennai - 600 036, India.

[4]Department of Chemistry, Khalifa University of Science and Technology, Abu Dhabi - 127788, United Arab Emirates.

[5]Center for Catalysis and Separations, Khalifa University of Science and Technology, Abu Dhabi - 127788, United Arab Emirates.

† Authors contributed equally.

[*] Correspondence:

Govindaraj Perumal - govindarajp.sdc@saveetha.com

Sreenath Pappuru - sreenath.technion@gmail.com

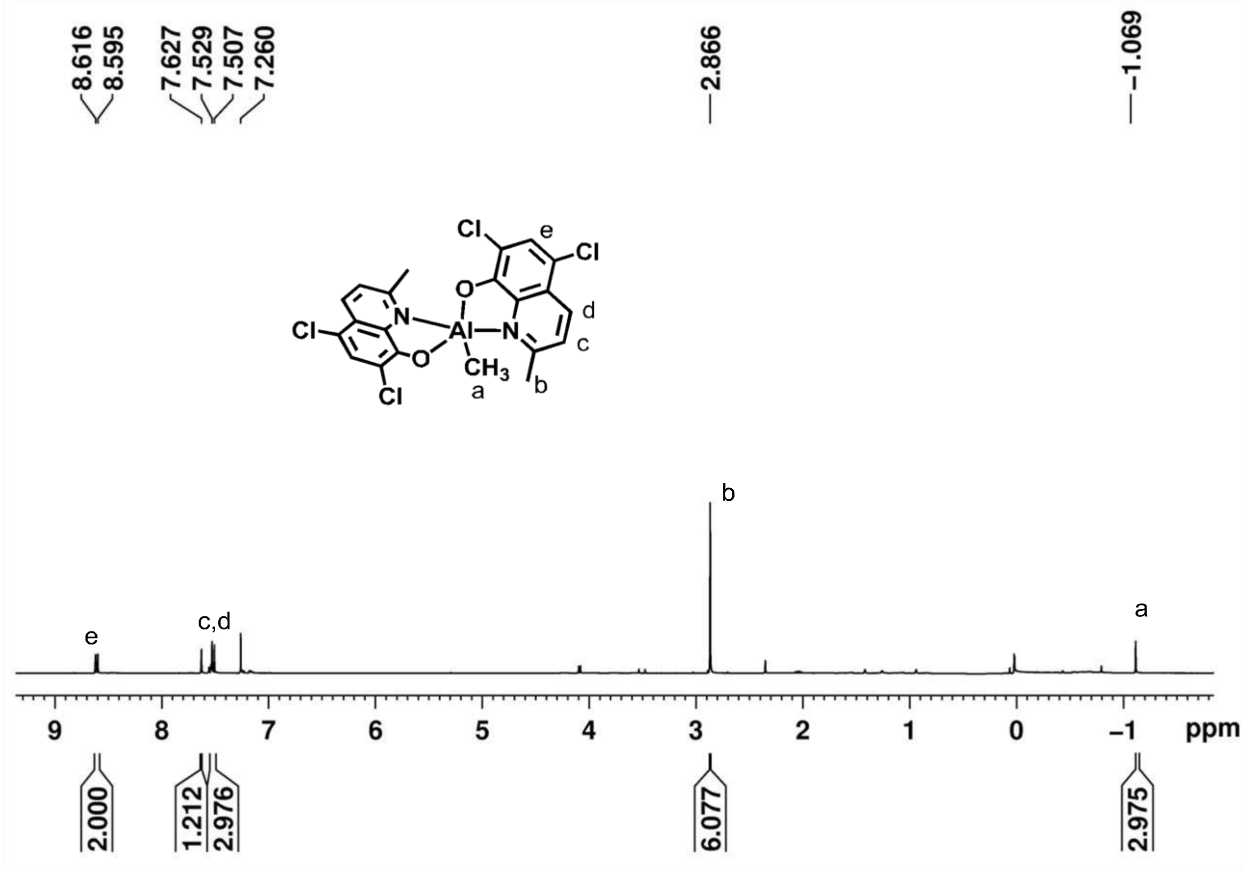

Figure S1: ^{1}H NMR (400 MHz, $CDCl_3$) of Al compound, **1**.

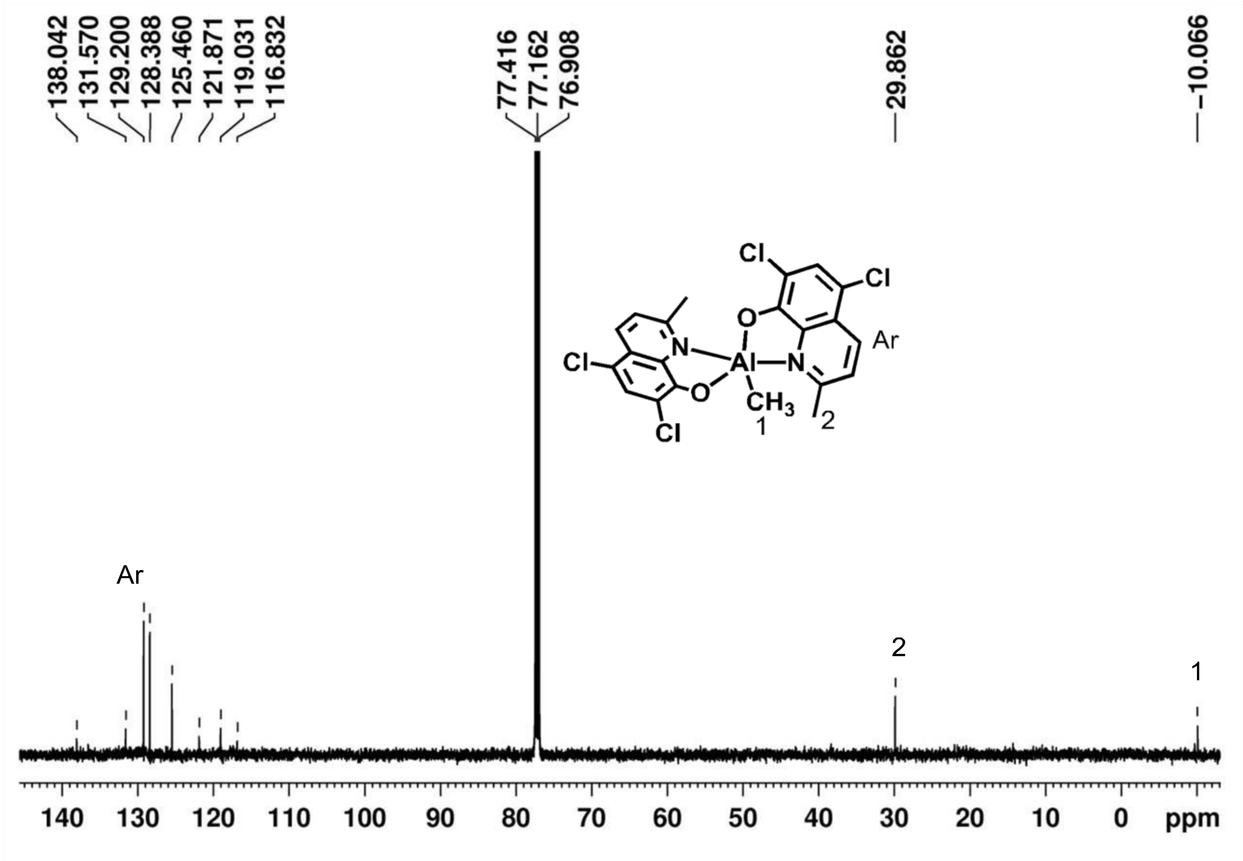

Figure S2: ^{13}C NMR (100 MHz, $CDCl_3$) of Al compound, **1**.

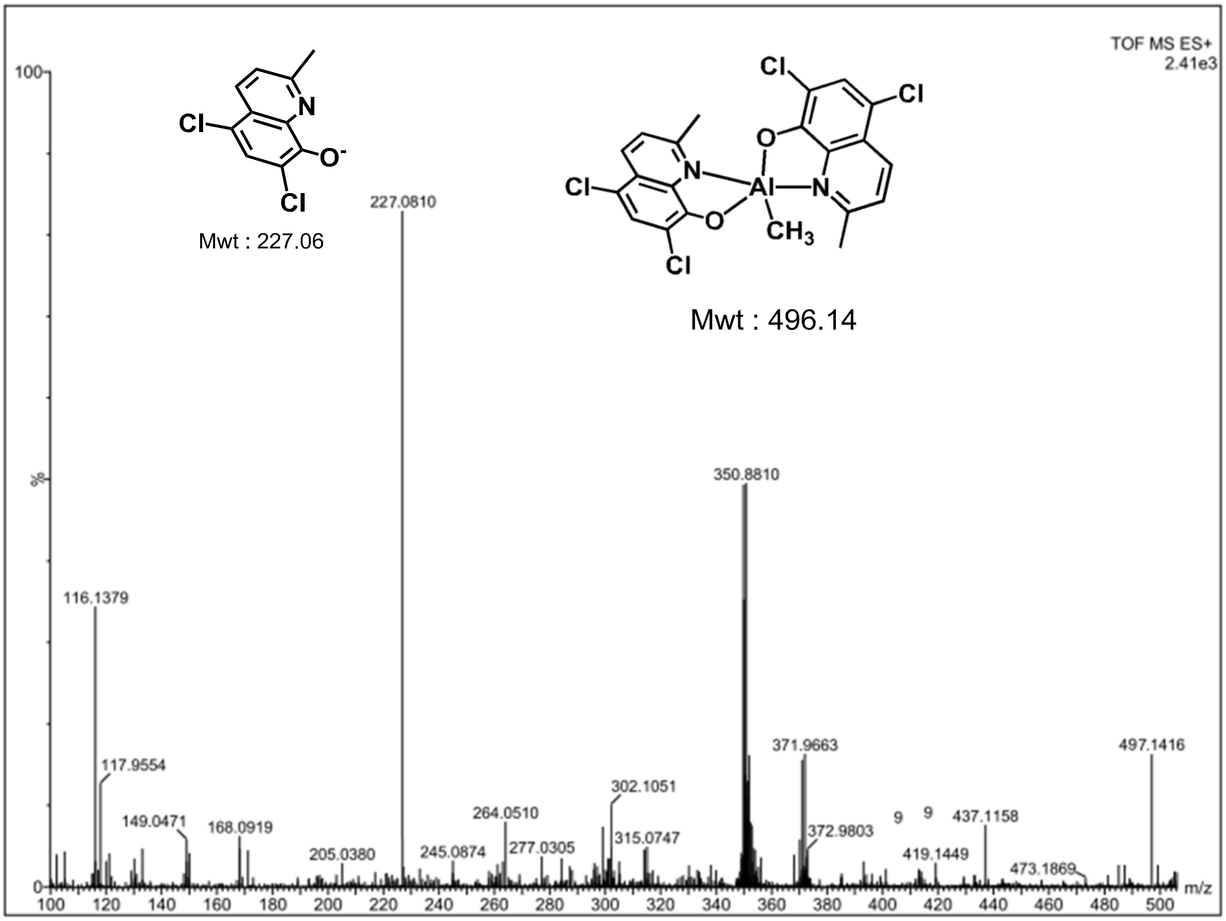

Figure S3: ESI-Mass Spectrum of Al compound, **1**.

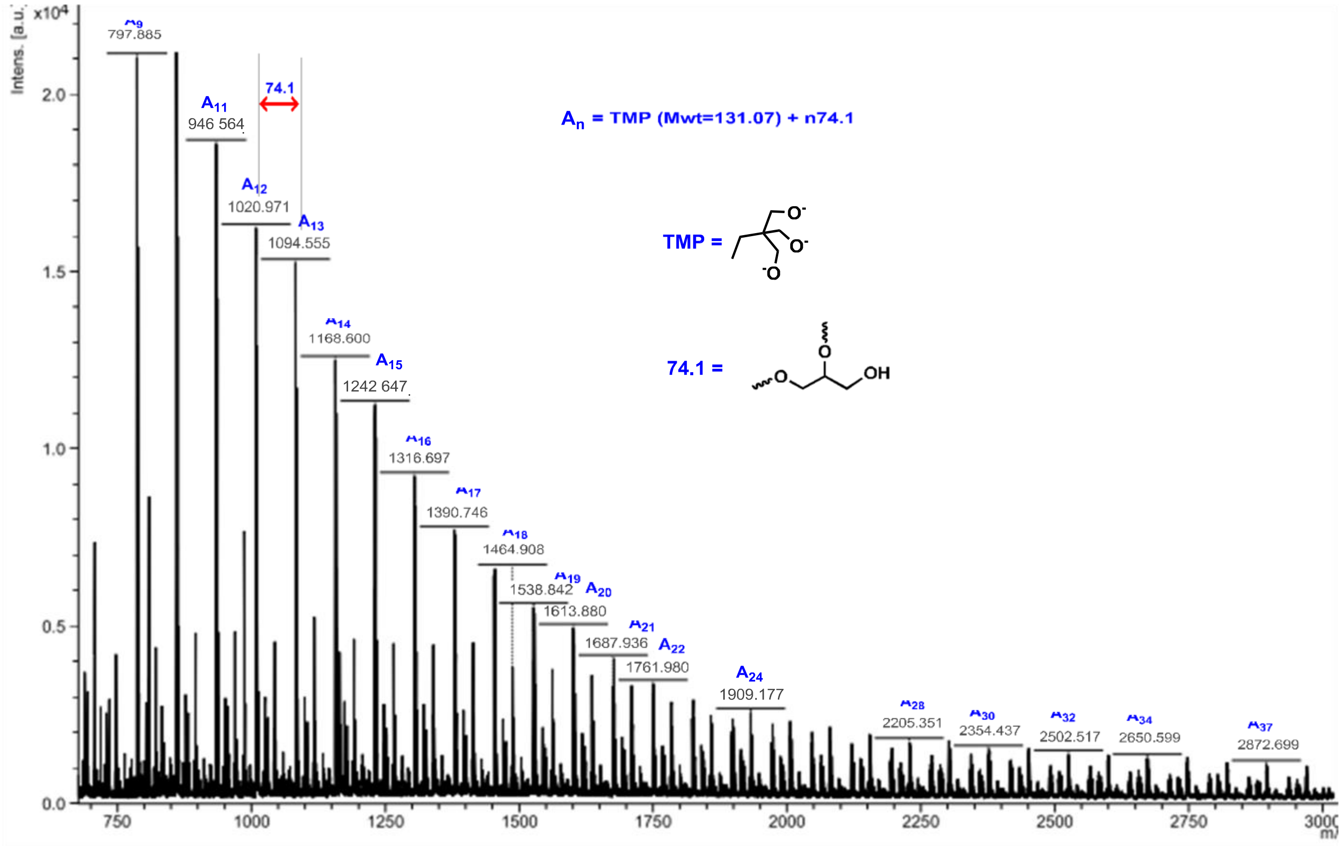

Figure S4: MALDI-TOF spectrum of low molecular weight HPG using aluminium complex as catalyst and TMP as initiator.

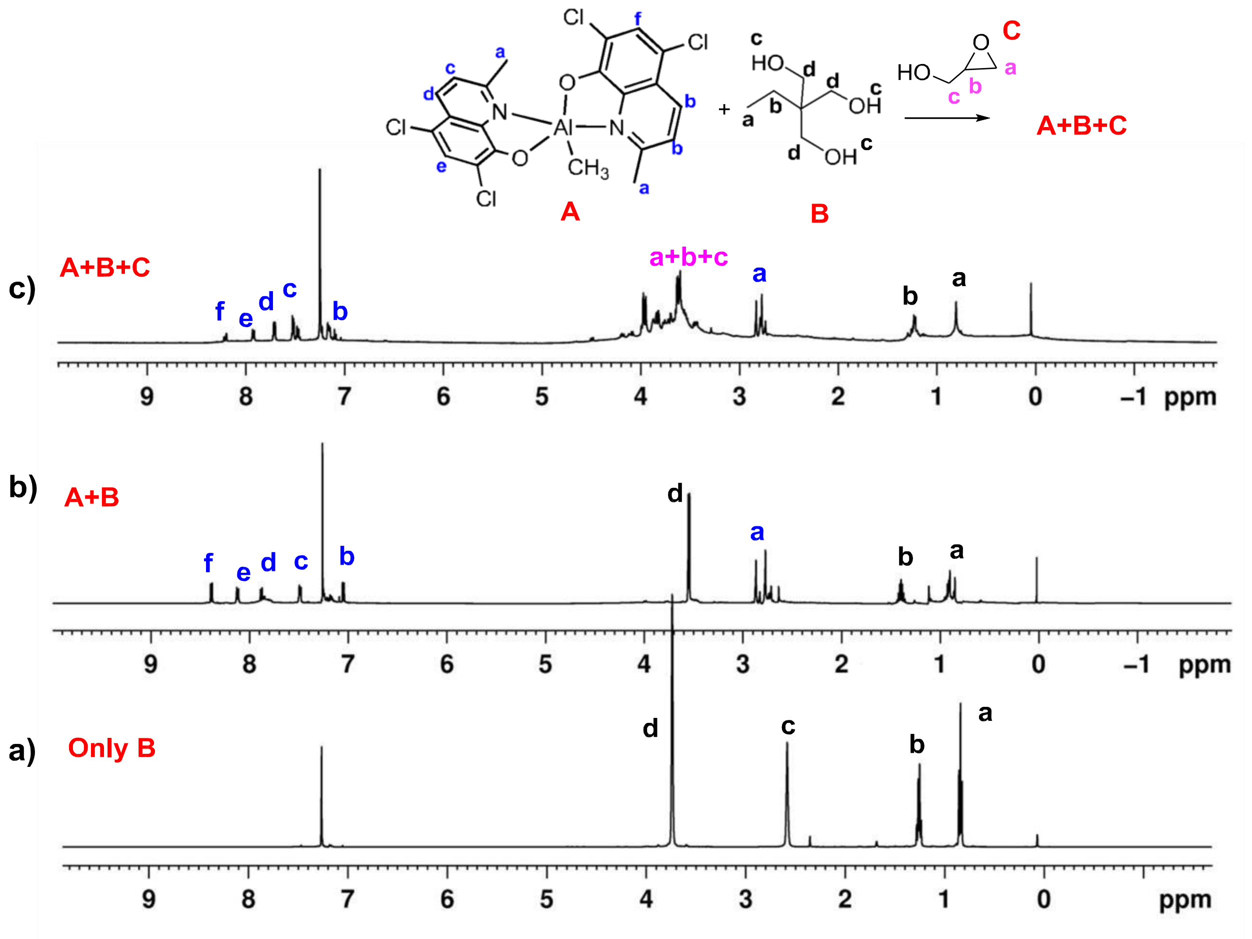

Figure S5. ^{1}H NMR spectra (400 MHz, $CDCl_3$) of only 1,1,1-Tris(hydroxymethyl)propane (TMP) initiator and TMP reacted aluminium complex in 1:1 molar ratio (a, b) and oligomer species prepared using TMP reacted aluminium complex (1equiv) and glycidol (5 equiv) in dry toluene at 95 ^{o}C, 2 h (c).

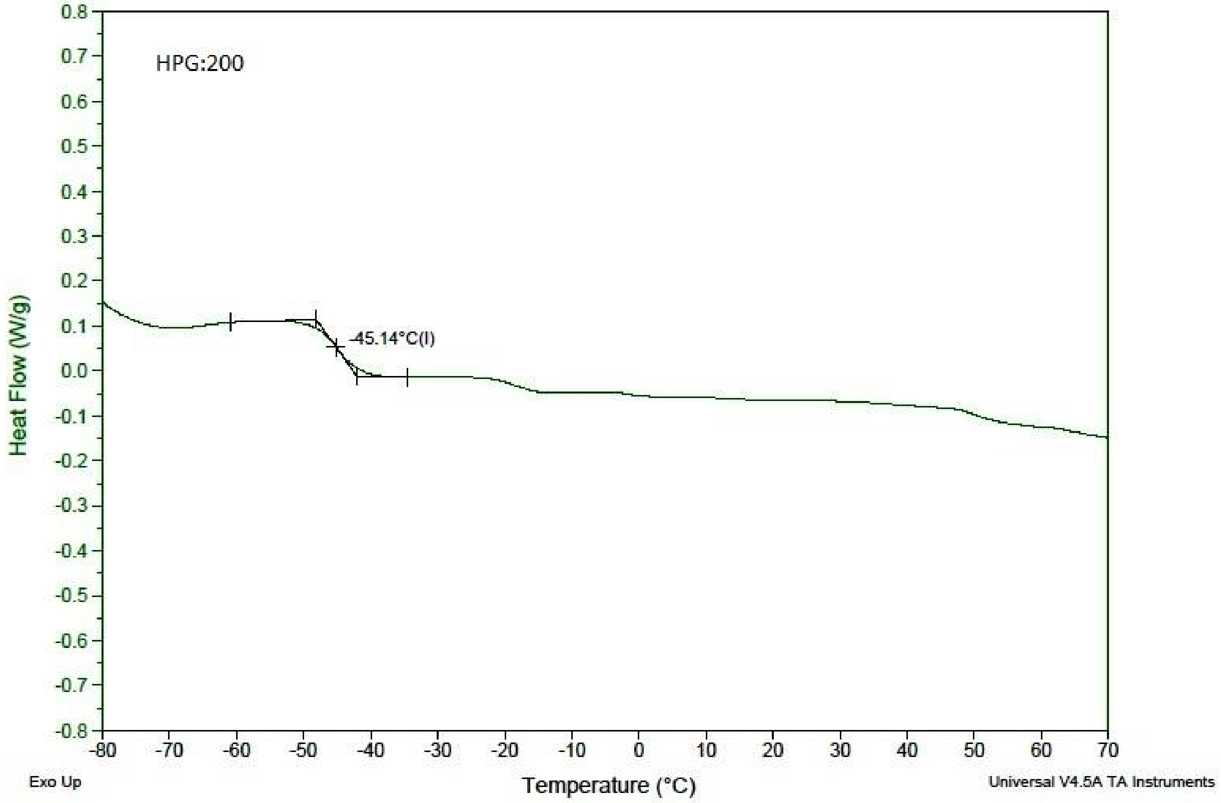

Figure S6: DSC thermogram of HPG 200

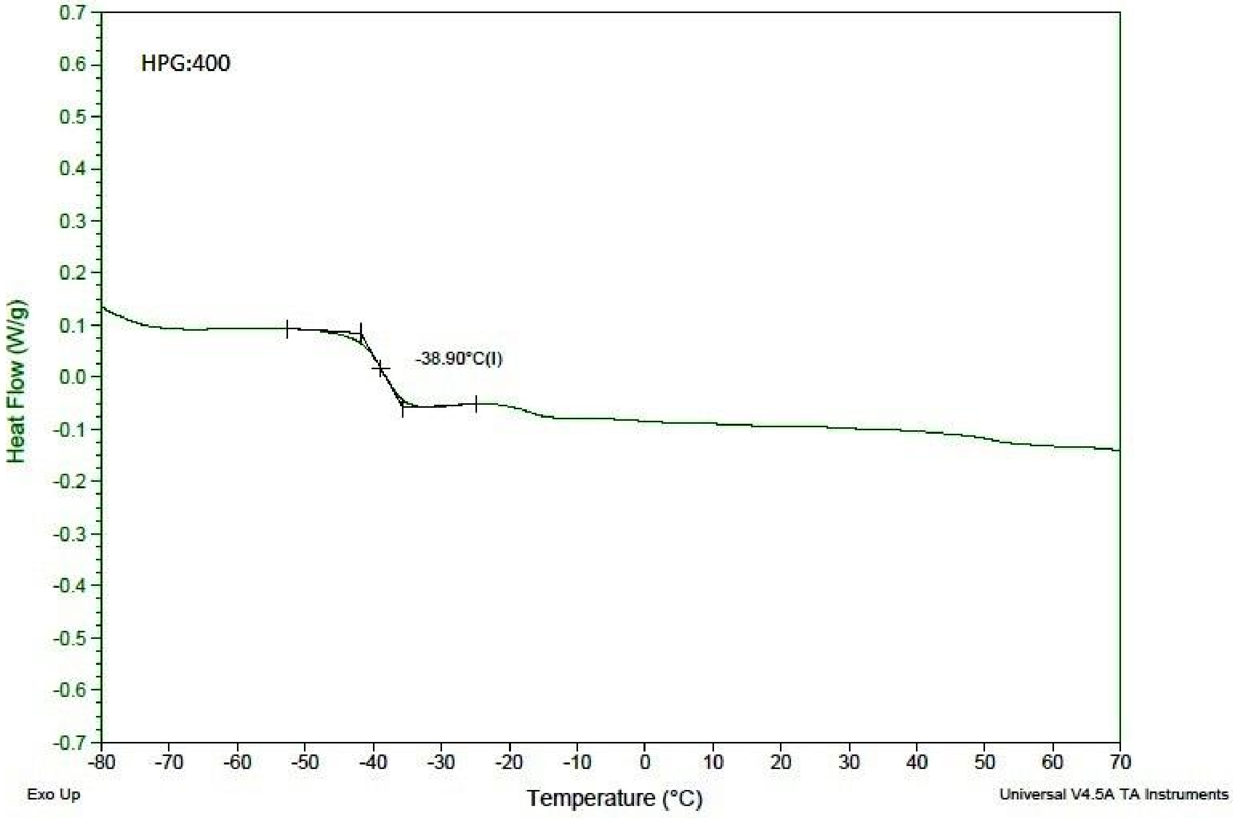

Figure S7: DSC thermogram of HPG 400

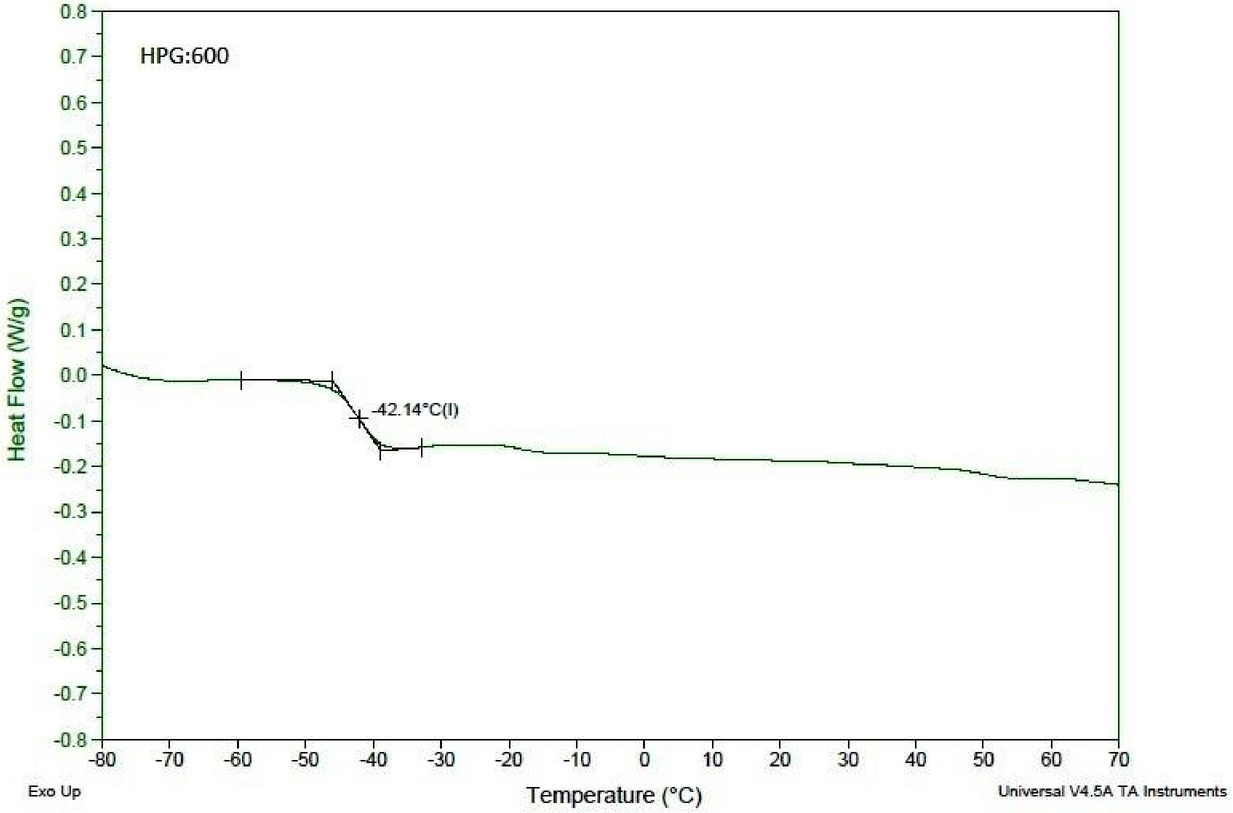

Figure S8: DSC thermogram of HPG 600

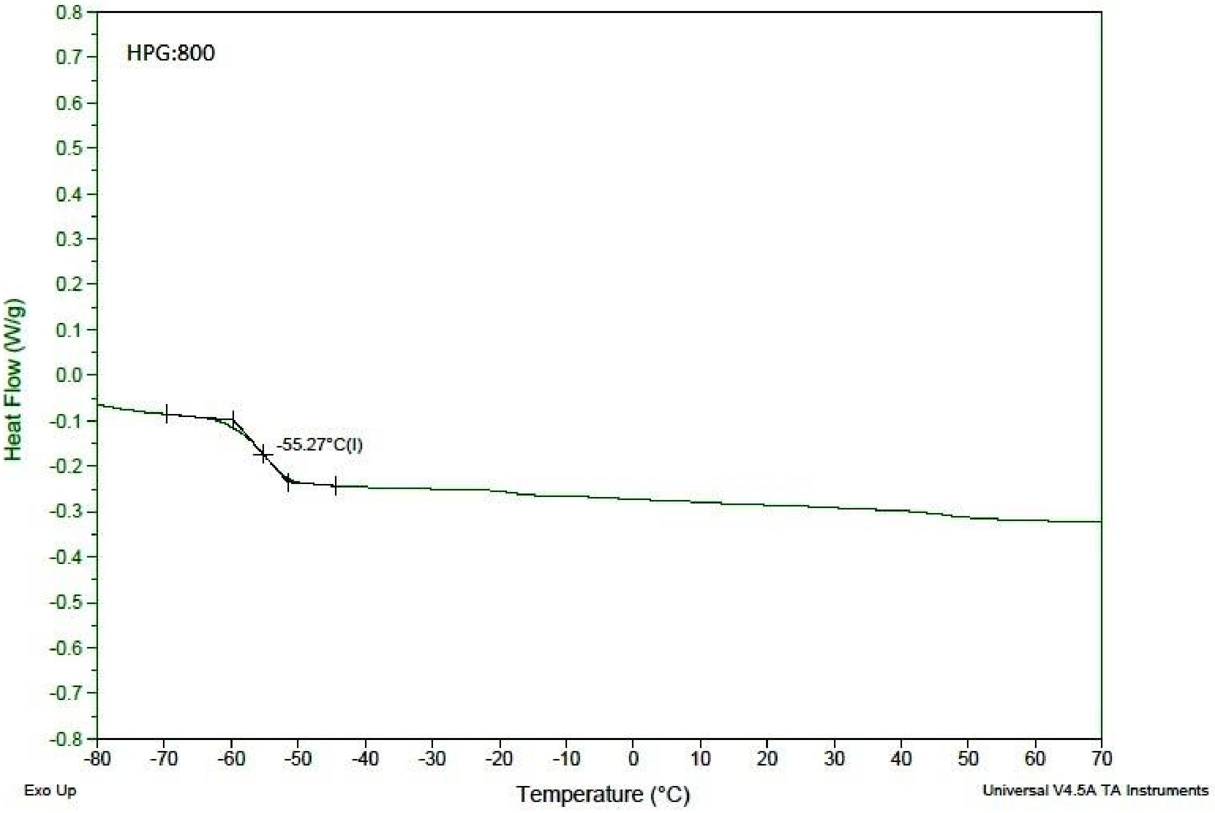

Figure S9: DSC thermogram of HPG 800

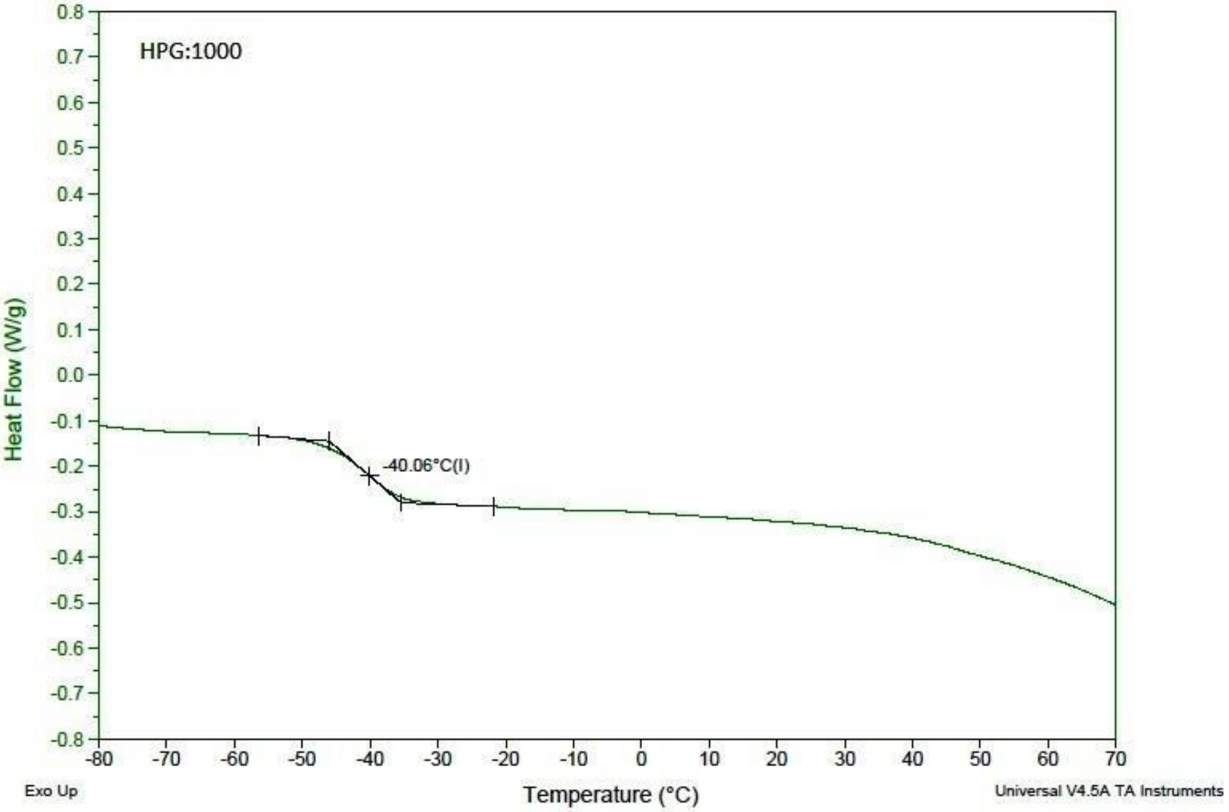

Figure S10: DSC thermogram of HPG 1000

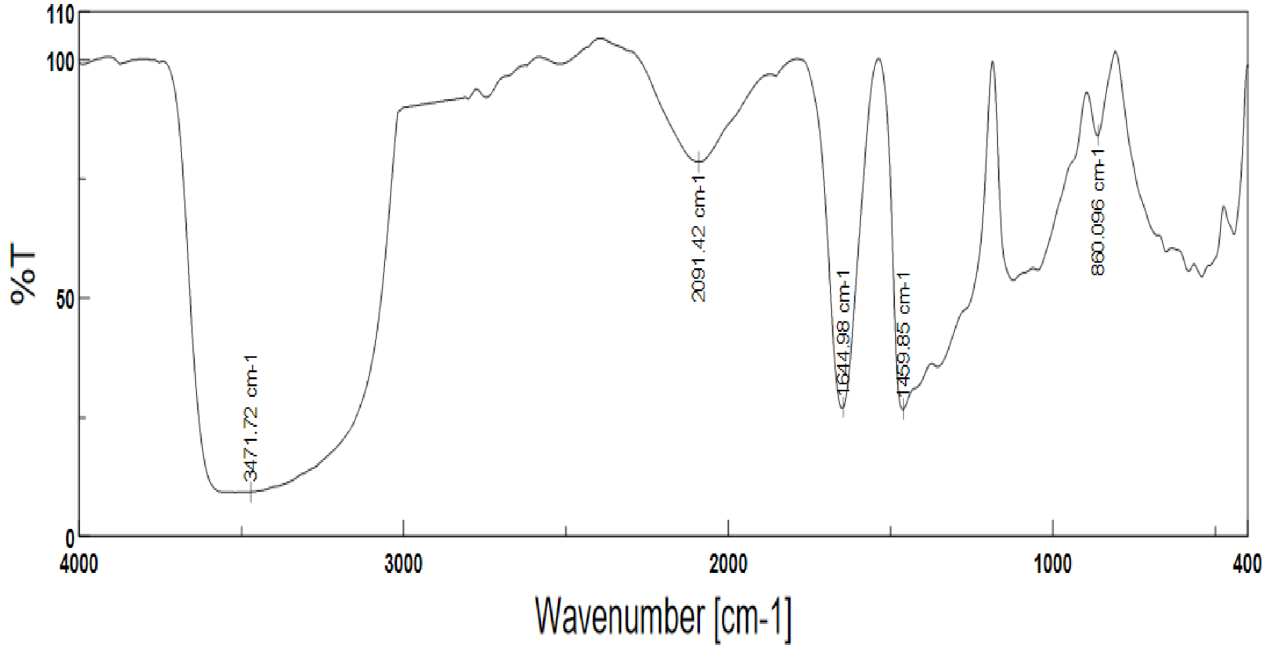

Figure S11: FT-IR spectrum of HPG 200

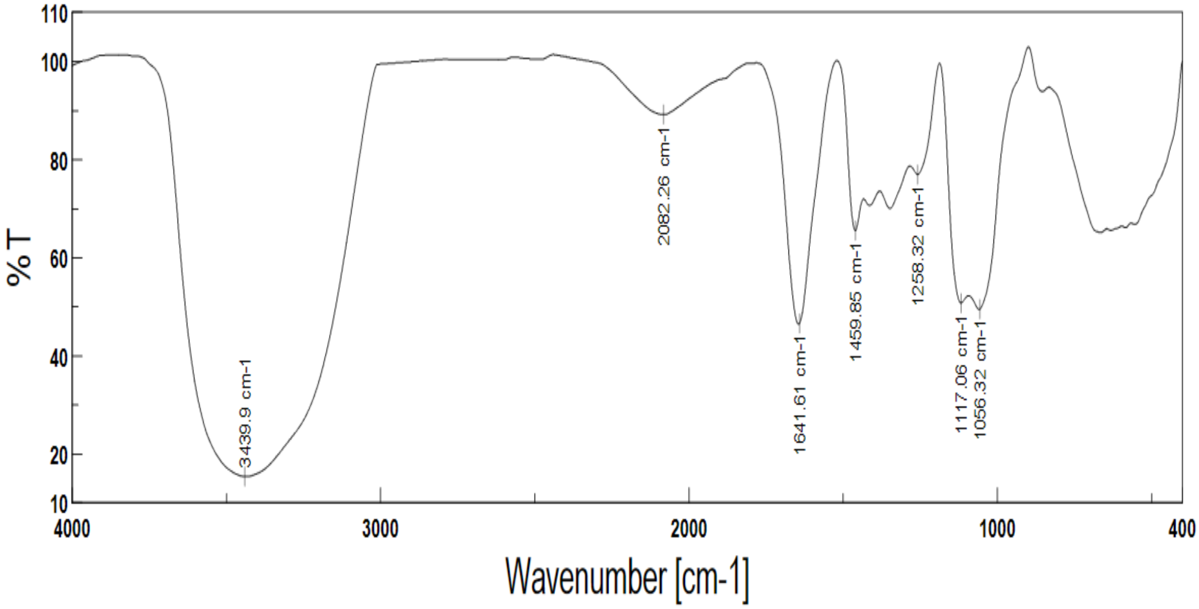

Figure S12: FT-IR spectrum of HPG 400

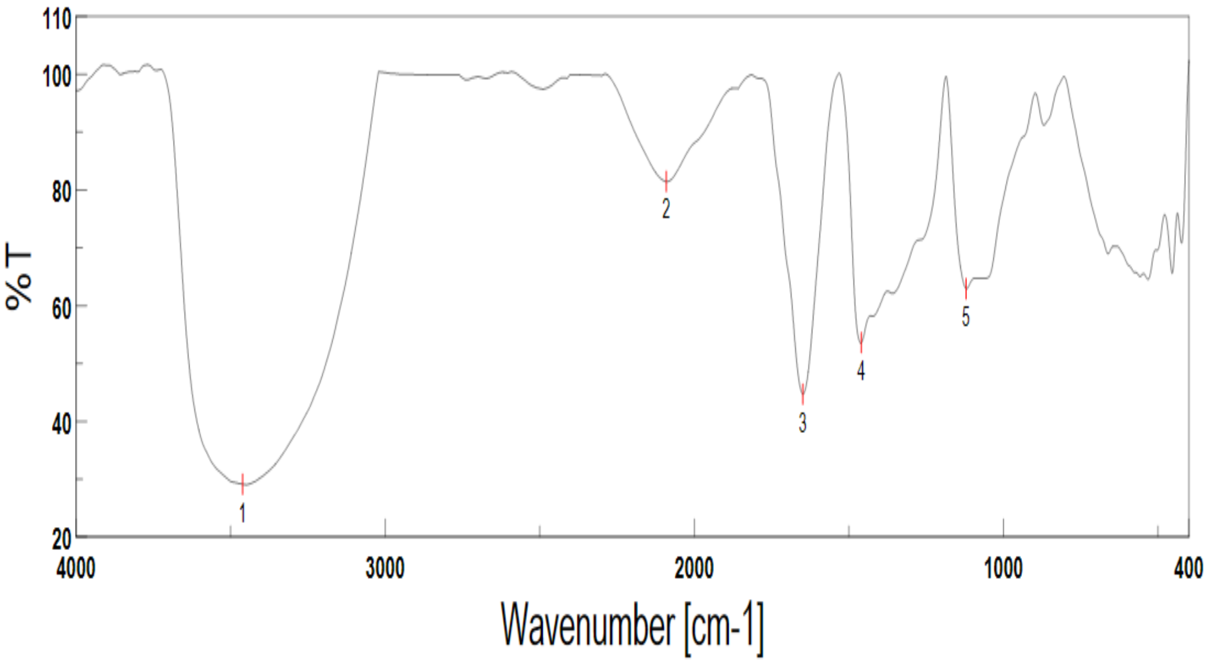

Figure S13: FT-IR spectrum of HPG 600

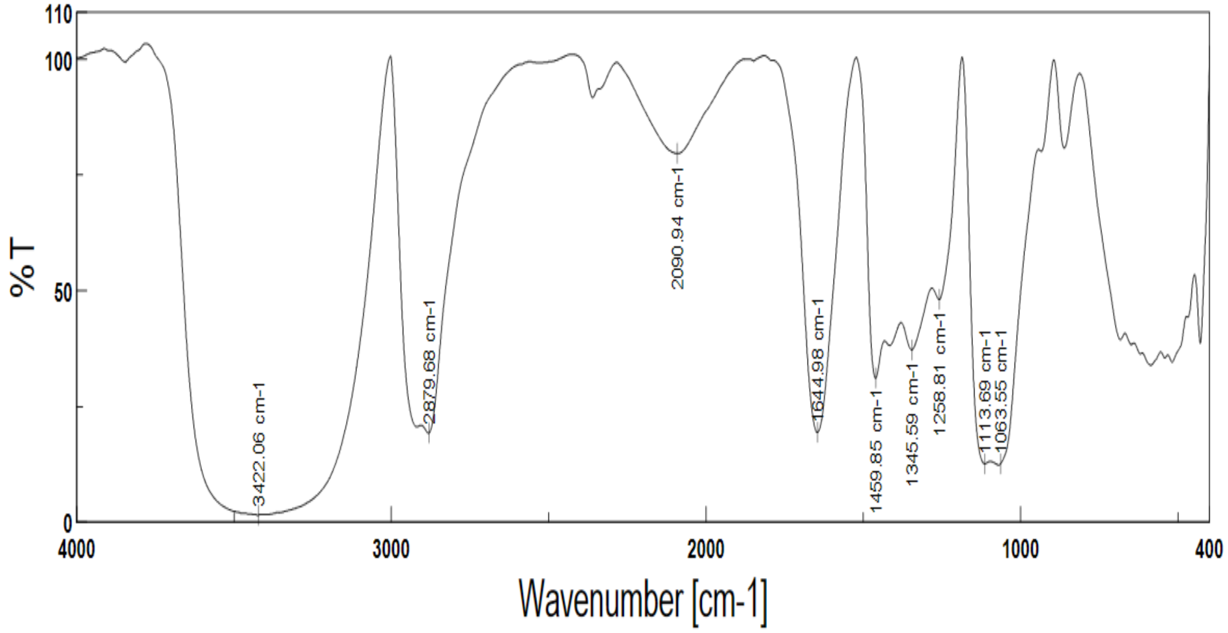

Figure S14: FT-IR spectrum of HPG 800

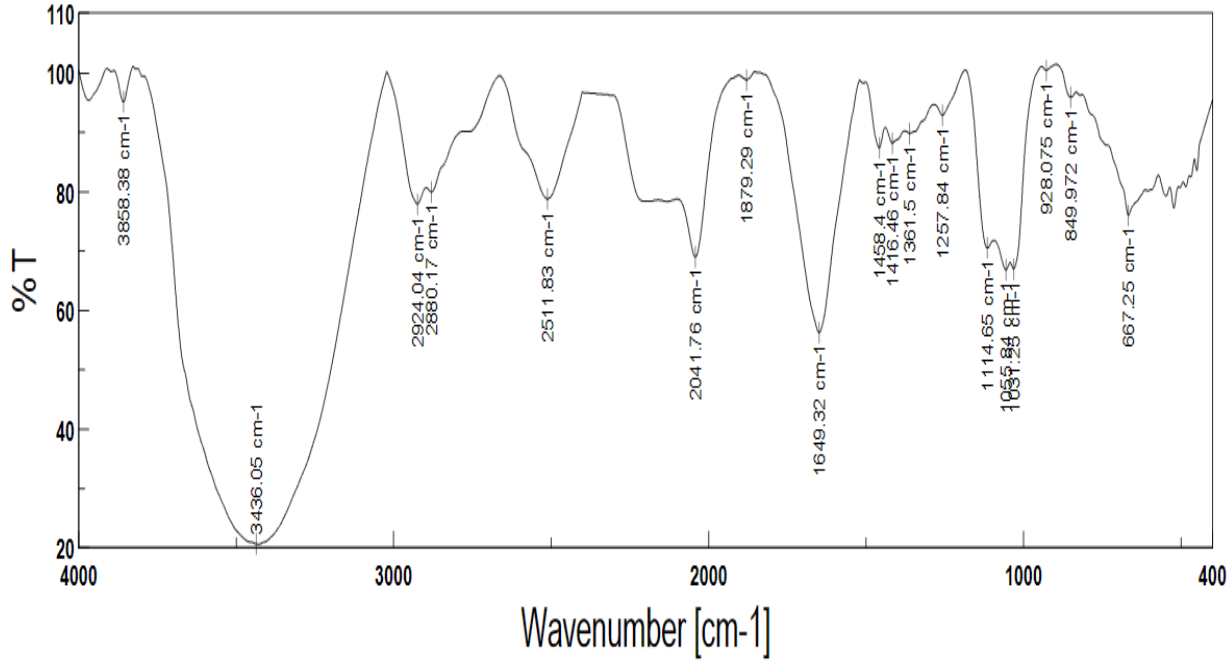

Figure S15: FT-IR spectrum of HPG 1000

Aluminium Complex **X-ray data**

Chemlcal formula	$C_{21}H_{15}AlCl_4N_2O_2$
Formula weight	496.13 g/mol
Temperature	296(2) K
Wavelength	0.71073 Å
Crystal system	trlcllnlc
Space group	P 21/n
Unlt cell dlmenslons	a = 24.283(3) Å α = 90°
	b = 7.3254(8) Å ß = 95.963(7) °
	c = 26.107(4) Å , γ = 90 °
Volume	4618.9(10) $Å^3$
Density (calculated)	1.427 g/cm^{-3}
Absorption coefflclent	0.571 mm^{-1}
Flnal R indices(I>2s(I))	R1 = 0.116, wR2 = 0.3665
